# Design of allosteric sites into rotary motor V_1_-ATPase by restoring lost function of pseudo-active sites

**DOI:** 10.1038/s41557-023-01256-4

**Published:** 2023-07-06

**Authors:** Takahiro Kosugi, Tatsuya Iida, Mikio Tanabe, Ryota Iino, Nobuyasu Koga

**Affiliations:** 1 Research Center of Integrative Molecular Systems (CIMoS), Institute for Molecular Science (IMS), National Institutes of Natural Sciences (NINS), Okazaki, Japan; 2 Exploratory Research Center on Life and Living Systems (ExCELLS), National Institutes of Natural Sciences (NINS), Okazaki, Japan; 3 Department of Structural Molecular Science, School of Physical Sciences, SOKENDAI (The Graduate University for Advanced Studies), Hayama, Japan; 4 PRESTO, Japan Science and Technology Agency, Kawaguchi, Japan; 5 Department of Life and Coordination-Complex Molecular Science, Institute for Molecular Science (IMS), National Institutes of Natural Sciences (NINS), Okazaki, Japan; 6 Department of Functional Molecular Science, School of Physical Sciences, SOKENDAI (The Graduate University for Advanced Studies), Hayama, Japan; 7 Structural Biology Research Center, Institute of Materials Structure Science, High Energy Accelerator Research Organization (KEK), Tsukuba, Japan; 8 Present Address: Institute for Protein Research (IPR), Osaka University, Suita, Japan

**Keywords:** Enzyme mechanisms, Biophysical chemistry, Protein design, Motor protein regulation

## Abstract

Allostery produces concerted functions of protein complexes by orchestrating the cooperative work between the constituent subunits. Here we describe an approach to create artificial allosteric sites in protein complexes. Certain protein complexes contain subunits with pseudo-active sites, which are believed to have lost functions during evolution. Our hypothesis is that allosteric sites in such protein complexes can be created by restoring the lost functions of pseudo-active sites. We used computational design to restore the lost ATP-binding ability of the pseudo-active site in the B subunit of a rotary molecular motor, V_1_-ATPase. Single-molecule experiments with X-ray crystallography analyses revealed that binding of ATP to the designed allosteric site boosts this V_1_’s activity compared with the wild-type, and the rotation rate can be tuned by modulating ATP’s binding affinity. Pseudo-active sites are widespread in nature, and our approach shows promise as a means of programming allosteric control over concerted functions of protein complexes.

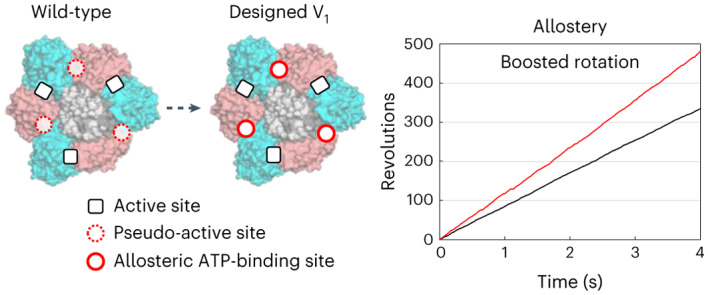

## Abstract (web summary)

Allostery produces concerted functions of protein complexes by orchestrating the cooperative work between the constituent subunits. By restoring functions of pseudo-active sites that have been lost through evolution, allosteric sites have now been designed into a rotary molecular motor, V_1_-ATPase, resulting in its rotation being boosted allosterically.

## Main

Protein complexes such as haemoglobin and molecular motors exert concerted functions through cooperative work between the subunits^1,2^. The orchestration between the subunits is enabled by the allosteric mechanism, which involves functions exerted at an active site in a subunit being regulated by binding of an effector molecule to an allosteric site in another subunit^1^. The creation of artificial allosteric sites in such protein complexes would provide the principles for allostery and potential tools for synthetic biology. Here we describe an approach to create allosteric sites in protein complexes.

**Fig. 1:**
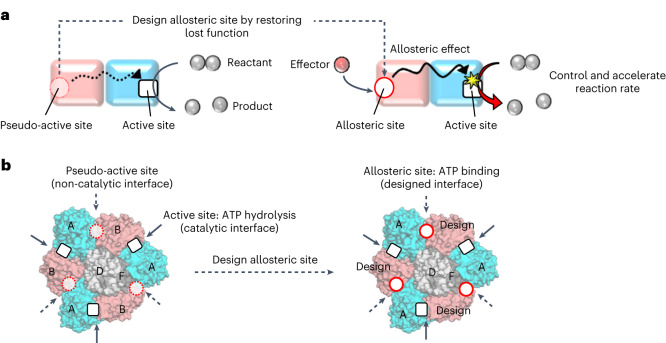
Strategy for designing allosteric sites in protein complexes. **a**, Strategy for the design of an allosteric site in a protein dimer complex. The non-catalytic subunit (pink) has a pseudo-active site, the function of which (for example, the ability to bind to a small molecule) has been lost during evolution. By computationally restoring the lost function, this pseudo-active site is engineered to be an allosteric site that controls a function of the active site in the catalytic subunit (cyan) upon the small (that is, effector) molecule binding. **b**, Overview of design of allosteric sites in *E. hirae* V_1_-ATPase. The active sites in the catalytic interface and the pseudo-active sites in the non-catalytic interfaces are indicated by solid and dashed arrows, respectively, in the hexameric ring of V_1_ consisting of A subunits (cyan) and B subunits (pink). The rotor of the D and F subunits (grey) is located in the centre of the ring. The pseudo-active sites in the non-catalytic interfaces are designed to be ATP-binding allosteric sites that control the ATP hydrolysis function of the active sites in the catalytic interfaces.

Various protein complexes include subunits that have pseudo-active sites, the functions of which are assumed to have been lost during evolution^3–6^. Pseudo-active sites have been suggested to have an allosteric connection with active sites in other subunits^3–5^. For example, a pseudo-active site in a subunit that has lost ATPase activity but still exhibits ATP-binding ability could activate another subunit’s active site upon binding to ATP^7,8^. These studies support the hypothesis that distinct allosteric sites can be created in protein complexes by engineering pseudo-active sites, that is, restoring their lost functions (Fig. 1a).

The protein complex rotary motor V_1_-ATPase (V_1_) contains a pseudo-active site in a constituent subunit. V_1_ is a part of an ion pump V-ATPase, which transports cations across the membrane by ATP-hydrolysis-driven rotation^9^. V_1_ comprises a rotor composed of the D and F subunits, and a stator, a hexameric ring, composed of three A subunits and three B subunits (Fig. 1b)^10^. The B subunit has homology with the A subunit (for example, for *Enterococcus hirae* V_1_-ATPase, the BLAST E-value between the subunits is 4 × 10^−22^) and the overall subunit structures resemble each other (Supplementary Fig. 1). However, the details of their sequences and structures are different, resulting in two different interfaces between the A and B subunits in the A_3_B_3_ hexameric ring: a catalytic interface, at which the A subunit has an ATP-hydrolysis catalytic site; and a non-catalytic interface, at which the B subunit has a pseudo-active site, which has evolutionally lost the ATP-hydrolysis or even binding ability (Fig. 1b)^10–12^. We test our hypothesis by restoring the lost ATP-binding ability of the pseudo-active site in the B subunit of *E. hirae* V_1_-ATPase through computational design.

## Results

### Computational design of an ATP-binding allosteric site

**Fig. 2:**
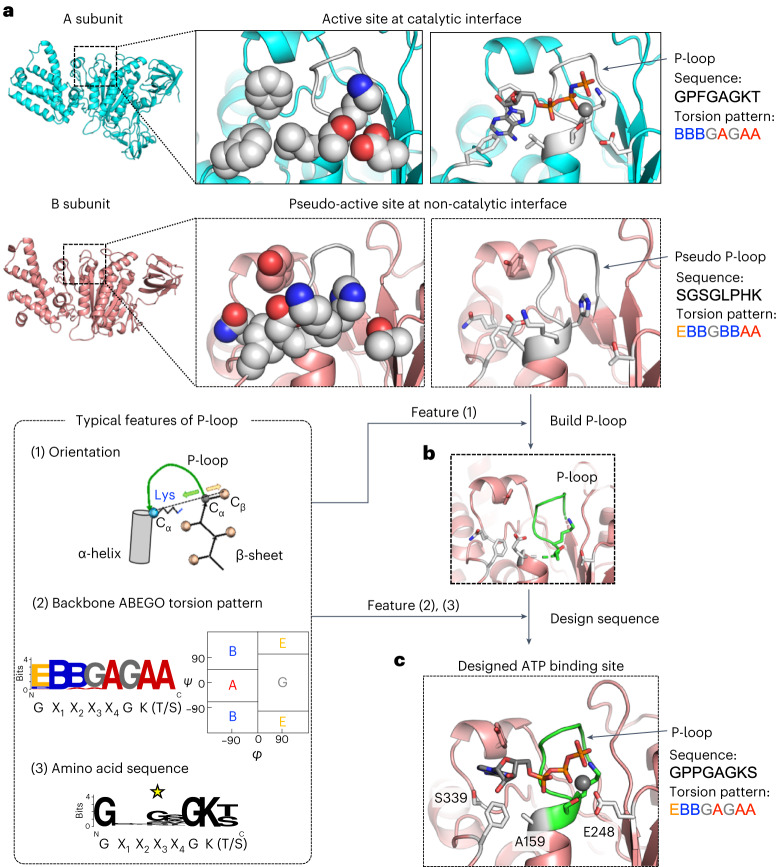
Computational design of an ATP-binding allosteric site by engineering the pseudo-active site of V_1_-ATPase. **a**, Structural differences between the active site in the A subunit and the pseudo-active site in the B subunit. The A subunit’s active site has a P-loop (GX_1_X_2_X_3_X_4_GK(T/S)), which is a well-known motif for binding to the phosphate group of ATP, and has a space to bind ATP. On the other hand, the B subunit’s pseudo-active site does not have either the P-loop or space for ATP binding. The B subunit has a loop (that is, the pseudo P-loop) at the position corresponding to the P-loop in the A subunit. The amino acid sequence and backbone geometry of the pseudo P-loop are different from the typical ones of P-loops, and the space around the pseudo P-loop is filled with side chains. **b**, A backbone structure of the P-loop built at the pseudo-active site. **c**, The created ATP-binding site at the pseudo-active site. Residues of the built P-loop (green) and grey residues (11 positions in total) were selected for the side-chain design. The residues changed from the original sequence by the design are denoted with characters: the P-loop was built at the residue positions 151–158 with the amino acid sequence GPPGAGKS; the Walker-B motif coordinating magnesium ion was built with Glu248; the nucleotide-binding pocket was made with Ala159 and Ser339. The typical features of P-loops are shown at the left bottom. Orientation: the vector from the C_α_ atom of the last strand residue immediately before the P-loop to the C_α_ atom of the conserved Lys points away from the vector from the C_α_ atom of the same last strand residue to its C_β_ atom. Backbone torsion pattern: the residues in P-loops have the typical backbone torsion pattern, represented by ABEGO^31^ torsion bins in conformational space defined using φ/ψ backbone dihedral angles: EBBGAGAA (the torsion bins A and B are the α-helix and β-sheet regions; G and E are the positive φ regions). Amino acid sequence: the P-loop is identified by the conserved sequence GX_1_X_2_X_3_X_4_GK(T/S). In addition, this motif has an additional conserved residue Gly at X_3_, indicated by a star. The torsion pattern and amino acid sequence logos were created by WebLogo^32^.

The active site in the A subunit has the well-known loop motif for binding to the phosphate group of ATP, which is commonly called the Walker-A motif (GX_1_X_2_X_3_X_4_GK(T/S))^13,14^ or P-loop, whereas the pseudo-active site in the B subunit does not have the P-loop (Fig. 2a), but instead has a loop at the position corresponding to the P-loop of the A subunit (we term this loop the pseudo P-loop). The amino acid sequence and backbone torsion pattern of the pseudo P-loop are completely different from the typical ones of P-loops (Fig. 2, left bottom). Moreover, the pseudo-active site is filled with side chains and does not have a space for binding of an ATP molecule (Fig. 2a). Recently, computational methods for designing small molecule binding sites have been developed using Rosetta design software^15–17^. We computationally redesigned the pseudo P-loop and its surrounding residues to create an ATP-binding allosteric site (catalytic residues for ATP hydrolysis were not designed).

First, the backbone structure of a P-loop was built at the pseudo-active site in the B subunit by using the backbone of the A subunit’s P-loop (Fig. 2b), considering the orientation (Fig. 2, left bottom (1)). Subsequently, amino acid sequences and their side-chain conformations were designed on the P-loop backbone structure and the surrounding residues to stabilize the P-loop conformation in the designed site and have favourable interactions with an ATP molecule. In this design procedure, the conserved amino acid at X_3_ in native P-loops, Gly, was used in the designed P-loop (Fig. 2, left bottom (3))^18^, and favourable ATP binding modes were explored using multiple ATP conformations and constraints for the typical distances between the atoms of the P-loop and the phosphate atoms of ATP (Supplementary Fig. 2). The designed positions were limited to as few residues as possible to avoid impairing the hexameric ring complex formation ability (the complex was broken when more mutations are introduced; Supplementary Figs. 3 and 4). The resulting designed structures bound to an ATP were energetically minimized. The sequence design followed by the energy minimization was iterated, and the designs with high ATP-binding ability predicted by the Rosetta score were selected; the designs that did not have the conserved backbone torsion pattern of native P-loops (Fig. 2, left bottom (2)) after the energy minimization were discarded. The ATP-binding abilities of 29 selected designs were further evaluated by short (10 ns) molecular dynamics simulations in the monomeric state (Supplementary Fig. 5). Finally, a designed V_1_ (Fig. 2c) was experimentally characterized. Details of the design procedure are described in Fig. 2, Methods and Supplementary Fig. 6.

### The designed B subunit binds to nucleotide

**Fig. 3:**
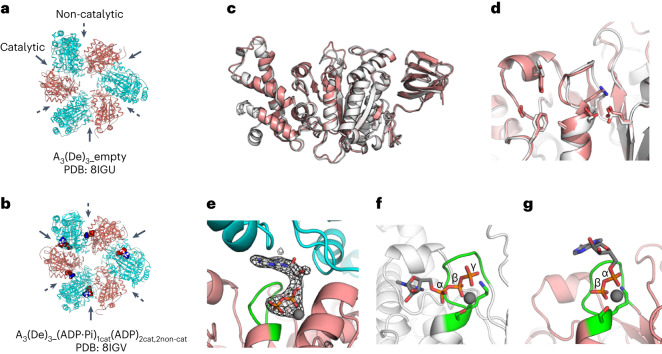
Nucleotide binding to the designed sites, revealed by crystal structures. **a**,**b**, Solved crystal structures viewed from the C-terminal domain of the A subunit and the designed B subunit: A_3_(De)_3_ complex structure in the absence of nucleotides (2.8 Å resolution) (**a**); A_3_(De)_3_ complex structure bound to three ADPs in the catalytic interfaces and two ADPs in the designed non-catalytic interfaces (3.2 Å resolution) (**b**). ADP molecules are shown as spheres. **c**,**d**, Superposition of the computational design model of the designed B subunit (white) and the solved crystal structure in the absence of nucleotides (pink) (chain D of A_3_(De)_3__empty was used because this had the highest resolution data of the three designed B subunits): comparison for the entire backbone (the C_α_ r.m.s.d. calculated by MICAN^33^ is 1.48 Å) (**c**); comparison for the designed binding site (**d**). The backbone geometry of the designed P-loop was almost identical to that of the design model. **e**, The interface between chains C and D in A_3_(De)_3__(ADP·Pi)_1cat_(ADP)_2cat,2non-cat_. The designed P-loop (green) firmly grabs the phosphate group of ADP. The sugar and base of ADP were found at the interface with the A subunit (cyan). The F_o_ − F_c_ omit map at 3.0*σ* obtained by removing ADP and Mg^2+^ from the model is shown in mesh representation. A stereo view is shown in Supplementary Fig. 8. **f**,**g**, Structural comparison for the ATP-binding mode between the computational design model (**f**) and the solved crystal structure (**g)** (chain D in A_3_(De)_3__(ADP·Pi)_1cat_(ADP)_2cat,2non-cat_). Oxygen, nitrogen and phosphate atoms are coloured red, blue and orange, respectively.

**Extended Data Fig. 1:**
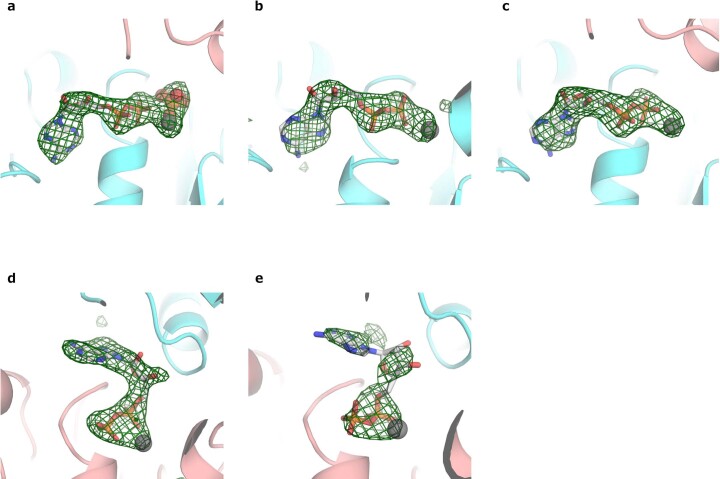
Fo-Fc omit maps for ligand molecules in A_3_(De)_3__(ADP·Pi)_1cat_(ADP)_2cat,2non-cat_. At the chain A (**a**), B (**b**), C (**c**), D (**d**), and E (**e**) in A_3_(De)_3__(ADP·Pi)_1cat_(ADP)_2cat,2non-cat_, the F_o_-F_c_ omit map at 3.0σ for ligand molecules are shown in green mesh. The A- and B-subunit are colored by cyan and pink, respectively.

The designed B subunit (De), expressed together with the A subunit in *Escherichia coli* and purified by Ni^2+^-affinity chromatography followed by gel-filtration chromatography, was found to form a ring complex with the A subunit (A_3_(De)_3_ ring complex) (Supplementary Fig. 7a). Subsequently, to evaluate the ATP-binding ability of De, we introduced a double mutation (K238A and T239A) in the A subunit to significantly impair its ATP-binding ability^19^. However, De did not form the A_3_(De)_3_ ring complex with the mutated A subunit (Supplementary Fig. 7b). Therefore, we determined the crystal structures of the A_3_(De)_3_ complex to prove the nucleotide-binding ability of De. First, the A_3_(De)_3_ complex was crystallized in the absence of nucleotide and the structure was solved at 2.8 Å resolution; this was named A_3_(De)_3__empty (Fig. 3a). The De structure in A_3_(De)_3__empty was almost identical to the computational model not only in terms of the overall structure but also in terms of the designed site together with the P-loop features (Fig. 3c,d). Next, the nucleotide-free crystals were incubated for 5 h in the presence of ADP by gradually increasing the ADP concentration to 10 mM, and the resulting structure, A_3_(De)_3__(ADP·Pi)_1cat_(ADP)_2cat,2non-cat_, was solved at 3.2 Å resolution. In A_3_(De)_3__(ADP·Pi)_1cat_(ADP)_2cat,2non-cat_, each of the three catalytic sites is occupied by an ADP (one of the sites has an ADP with a possible Pi) and each of the two designed sites out of the three is occupied by an ADP (Fig. 3b and Extended Data Fig. 1). This structure proved that the designed site has nucleotide-binding ability (Fig. 3e and Supplementary Fig. 8): the designed P-loop (green) exhibits typical backbone geometry (Fig. 2 left bottom) and accurately binds to the phosphate group of an ADP. However, interestingly, the ADP is bound in the opposite direction to the design (Fig. 3f,g): although the β phosphate is located at the same position as in the design model, the α phosphate is located at the position of the γ phosphate in the design model, and the sugar and base are at the interface with the A subunit. Note that, like naturally occurring P-loops, the designed P-loop grabs two phosphates. The observed unintended ATP-binding mode probably originated because we designed the binding site only in the monomer B subunit without considering the A subunit. The nucleotide-binding ability of the designed B-subunit monomer in solution was also indicated by thermal shift experiments^20^ in which we performed circular dichroism spectroscopy in the presence or absence of nucleotides (Supplementary Fig. 9); the dissociation constants of nucleotides against the designed B-subunit monomer were expected to be a few mM (we also carried out isothermal calorimetry and surface plasmon resonance measurements to determine the dissociation constants, but the values were too large to be determined by these methods). On the other hand, no ATP-hydrolysis activity of the designed B-subunit monomer was detected (Supplementary Fig. 10), which was expected because we did not design catalytic residues for ATP hydrolysis.

### The designed V_1_ rotates faster than the wild-type

**Fig. 4:**
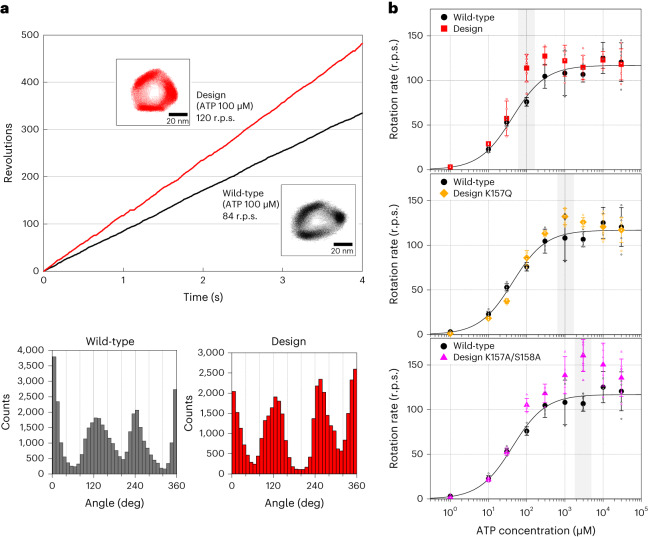
ATP binding to the designed site accelerates the rotation rate allosterically. **a**, A typical rotation time course of the designed V_1_ (red) and the wild-type V_1_ (black)^21^, at 100 µM ATP. All data for the wild-type V_1_-ATPase were obtained from ref. ^21^. The insets show the rotation *x*,*y* trajectory. The angle distributions are shown at the bottom. **b**, [ATP] dependence of rotation rates for the wild-type (black)^21^, the designed V_1_ (red), the design mutant K157Q (orange) and the design double-mutant K157A/S158A (magenta). The [ATP] at the most accelerated rotation is highlighted in grey. The rates were plotted with averaged values using three molecules or more (Supplementary Table 1) and the error bars represent the s.d. The black lines are the fitted curves to the Michaelis–Menten equation for the wild-type rotation rates. Source data

We carried out single-molecule experiments to observe the rotation of the A_3_(De)_3_DF complex (the A_3_(De)_3_ ring complex with a rotor DF) in various ATP concentrations ([ATP]s). The designed V_1_ was found to rotate unidirectionally in a counterclockwise fashion with discrete 120° steps, similar to the wild-type^21^, but rotates faster than the wild-type at 100 μM ATP (Fig. 4a). Moreover, in contrast to the wild-type, the rotation rates of the designed V_1_ were apparently not well-fitted to the Michaelis–Menten equation in a certain [ATP] range (Fig. 4b, top, and Supplementary Fig. 11). The rotation rates were similar to the wild-type at the lowest (1 μM) and highest (30 mM) [ATP]s, but the rotation was significantly accelerated in [ATP]s between several tens and hundreds of μM (Fig. 4b, top). At 100 μM ATP, the designed V_1_ showed the most accelerated rotation rate (113.9 ± 15.5 r.p.s.) compared with the wild-type (76 ± 4.8 r.p.s.) (Supplementary Fig. 12). We also measured ATPase activity of the wild-type and designed V_1_ by a conventional biochemical method. The ATPase activity of the designed V_1_ in this method also accelerated in a certain range of [ATP]s (Supplementary Fig. 13).

We also found that the [ATP] at which the designed V_1_ shows maximal acceleration compared with the wild-type can be tuned by modulating the nucleotide-binding affinity of the designed site (Fig. 4b). We first created a mutant with K157Q (the mutation of the conserved Lys in the P-loop at the position 157 to Gln), which is expected to decrease the ATP-binding ability^22^ (see also the Rosetta ddG score calculations in Supplementary Table 2). The rotation rates of the K157Q mutant were not well-fitted to the Michaelis–Menten equation, similar to the design, but notably, the [ATP] at the most accelerated rotation shifted to 1 mM, and was much higher than that for the original design (Fig. 4b, middle). Furthermore, we created a double mutant with K157A/S158A to further decrease the binding affinity (note that the mutant is still expected to have the capability to bind ATP^19^) (Supplementary Table 2). The rotation rates of the double mutant were also not well-fitted to the Michaelis–Menten equation, but the [ATP] at the most accelerated rotation further shifted to 3 mM (Fig. 4b, bottom). The rotation rate at 3 mM ATP was the highest (161 ± 18 r.p.s.) among those for the wild-type, the original design and the mutants at any [ATP]s (Fig. 4b). The observed correlation between the nucleotide-binding affinity of the designed site and the [ATP] at the most accelerated rotation strongly suggests that the allosteric effect is produced by the nucleotide binding to the designed site.

### Allostery facilitates ADP release from the catalytic site

**Fig. 5:**
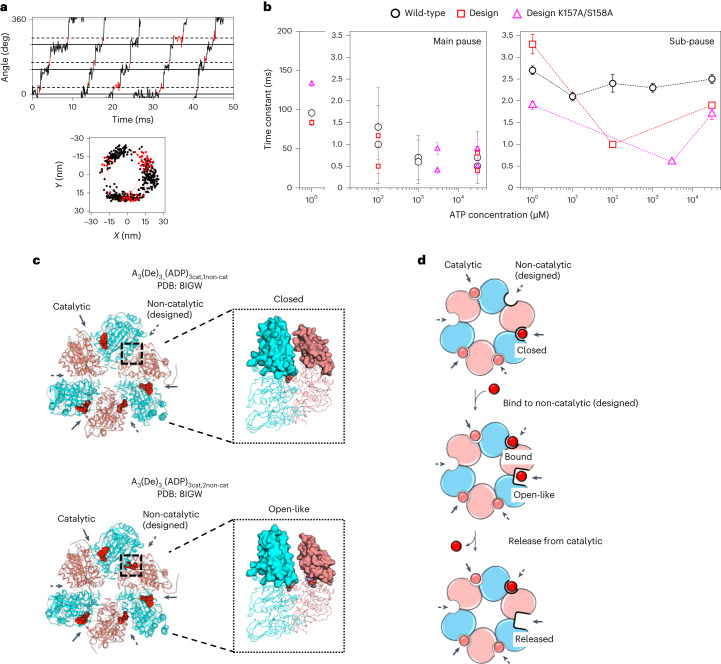
The mechanism of allosteric acceleration, revealed by analysis of rotation sub-steps and solved crystal structures. **a**, A close-up rotation time course of the design at 100 µM ATP and the rotation *x*,*y* trajectory. The main pause and sub-pause are shown in black and red, respectively. The black dashed and solid horizontal lines show angles for the main pause and sub-pause, respectively. **b**, Time constants of duration times of the main pause (left) and the sub-pause (right) at different [ATP]s for the wild-type V_1_ (black)^21^, the designed V_1_ (red) and the design double mutant K157A/S158A (magenta). The time constants were plotted with values obtained by analysing all detected pauses for three molecules and the error bars represent the s.d. See Supplementary Figs. 14 and 15 for distributions of the duration time. Note that for the main pauses at 100–3,000 μM ATP, two different time constants were obtained for each [ATP] assuming consecutive reactions (Supplementary Fig. 14). **c**, Comparison of A_3_(De)_3__(ADP)_3cat,1non-cat_ (top) and A_3_(De)_3__(ADP)_3cat,2non-cat_ (bottom). The hexameric structures viewed from the C-terminal domain of the A and B subunits (left) and the structures of the catalytic interfaces viewed from side (right) are shown for closed and open-like conformations, respectively. ADP molecules are shown as red spheres. **d**, Structure-based interpretation of the facilitated ADP release by the allosteric effect. Ellipses indicate the A subunits (cyan) and designed B subunits (pink). Nucleotides are shown by red (or pink) circles. Source data

To reveal the mechanism of allosteric acceleration, we investigated the single-molecule rotation in more detail. The designed V_1_ was found to have two sub-steps (40° and 80°) in the 120° step rotation (Fig. 5a and Supplementary Fig. 14), the same as the wild-type V_1_ (ref. ^21^). We analysed the duration times before the sub-steps at high and low [ATP]s (1 μM and 30 mM, respectively), in which the designed V_1_ rotated at a similar rate as the wild-type, and at 100 μM ATP, in which the design exhibited the most accelerated rotation. In the chemomechanical coupling model for the wild-type rotation^21^, the main pause corresponds to the duration time waiting for ATP binding, ATP hydrolysis and Pi release, and the sub-pause corresponds to that for ADP release. The main pause time constants for the design at each measured [ATP] are roughly the same as those of the wild-type, irrespective of [ATP] (Fig. 5b and Supplementary Fig. 14). The sub-pause time constants for the wild-type stayed constant between 2.1 and 2.7 ms at any [ATP]s, and the time constants for the design at the low and high [ATP]s were similar to those for the wild-type. However, the sub-pause time constant at 100 μM ATP, at which the rotation rate of the designed V_1_ was most accelerated, significantly decreased to 1.0 ± 0.02 ms (the time constant of the wild-type was 2.4 ± 0.2 ms) (Fig. 5b and Supplementary Figs. 14 and 16). The double mutant (K157A/S158A) also exhibited similar behaviour: the sub-pause time constant drastically decreased to 0.6 ± 0.002 ms at 3 mM ATP (the time constant of the wild-type was 2.5 ± 0.1 ms). These non-constant duration times for the sub-pause along with the [ATP] are the origin of the deviation of the fitting to the Michaelis–Menten equation (Fig. 4b). The rotation rates estimated from the measured time constants for the main pause and the sub-pause agreed with the observed rotation rates shown in Fig. 4b (Supplementary Table 3). All these results indicate that the origin of the accelerated rotation is the facilitation of ADP release at the catalytic sites, which is generated through the allosteric effect triggered by nucleotide binding to the designed sites.

**Extended Data Fig. 2:**
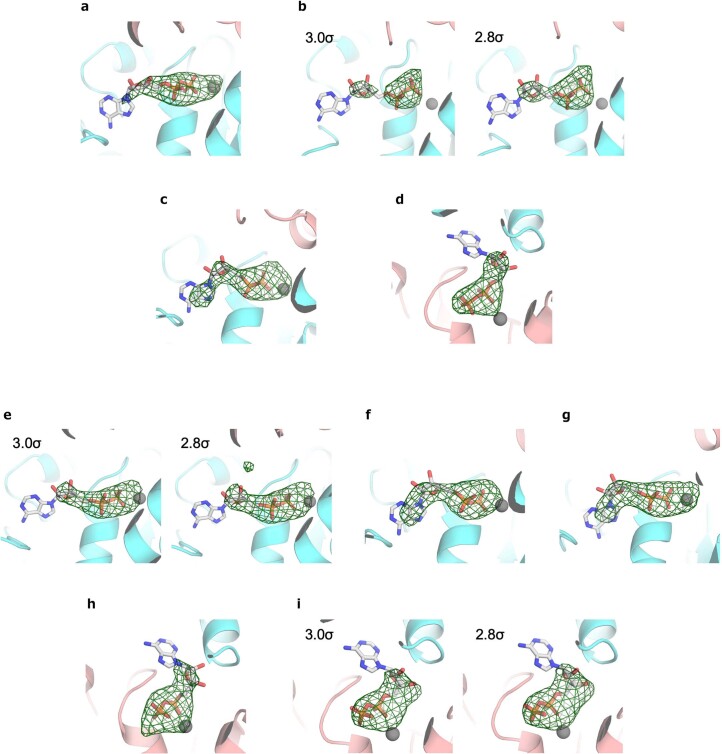
Fo-Fc omit maps for ligand molecules in A_3_(De)_3__(ADP)_3cat,1non-cat_ or A_3_(De)_3__(ADP)_3cat,2non-cat_. At the chain A (**a**), B (**b**), C (**c**), D (**d**), G (**e**), H (**f**), I (**g**), J (**h**), and L (**i**) in A_3_(De)_3__(ADP)_3cat,1non-cat_ or A_3_(De)_3__(ADP)_3cat,2non-cat_, the F_o_-F_c_ omit maps at 3.0σ for ligand molecules are shown in green mesh. The A- and B-subunit are colored by cyan and pink, respectively. For the chain B, G and L, the F_o_-F_c_ omit maps at 2.8σ are also shown to identify position of the molecules more clearly; the omit maps expand along the molecule shape by including the lower density value.

To provide a structure-based interpretation of the allosteric effect, two additional crystal structures were solved. The nucleotide-free crystals were incubated overnight by gradually increasing the ADP concentration to 5 mM, and two different structural states in an asymmetric unit were obtained from one dataset at 4.2 Å resolution. One is A_3_(De)_3__(ADP)_3cat,1non-cat_, in which each of the three catalytic sites and one of the three designed sites were occupied by an ADP (Fig. 5c, top, and Extended Data Fig. 2); the other is A_3_(De)_3__(ADP)_3cat,2non-cat_, in which each of the three catalytic sites and each of the two designed sites were occupied by an ADP (Fig. 5c, bottom, and Extended Data Fig. 2). Comparison of the two structures indicates that a nucleotide binding to the designed site induces the movement of the C-terminal α-helical domains of the designed B subunit and neighbour A subunit, which changes the conformation of the neighbouring catalytic interface from closed to open-like (Fig. 5c,d and Supplementary Table 4). The conformational change expands the space around the catalytic site formed between the α/β domains of the designed B subunit and neighbour A subunit (Supplementary Fig. 17). This probably weakens the interactions of the catalytic site with ADP. The conformational change suggests a structural mechanism for the facilitation of ADP release at the catalytic sites upon the ATP binding to the designed sites, although the structures are bound with ADP not ATP and do not contain the rotor subunits.

### Possible mechanism for allosteric acceleration

The [ATP] dependence of the rotation rate of the designed V_1_ can be explained as follows. At low [ATP], the allosteric effect does not emerge because ATP does not bind to the designed sites. For [ATP]s in the range from several tens to hundreds of μM, a nucleotide binding to one of the designed sites facilitates ADP release from the neighbouring catalytic site. The solved crystal structures suggest that the allosteric effect occurs upon the transition of the nucleotide occupation number for the designed sites from one to two (Fig. 5d). At high [ATP]s, the allosteric effect is suppressed, because the conformational transition when a nucleotide binds to the designed site (or the nucleotide binding itself) might be inhibited when the other two designed sites are occupied by ATPs. A theoretical model together with further rotational experiments and high-resolution structures is necessary to provide a more comprehensive explanation of the created allostery and rotation scheme.

It should be noted, in particular, that the three designed nucleotide-binding sites have different binding affinities because not all of them were occupied by nucleotide in the solved crystal structures. This would be crucial for allosterically accelerating the rotation rate: just as the nucleotide-binding affinity of each catalytic site changes during rotation, so the nucleotide-binding affinity for each allosteric site should also change during rotation to produce the allostery. The molecular mechanisms encoding such asymmetric and dynamically varying binding affinities have yet to be elucidated.

## Discussion

We succeeded in designing allosteric sites in a protein complex, the molecular motor V_1_-ATPase, by restoring the lost ATP-binding ability of the pseudo-active site in the B subunit. The allosteric mechanism was originally described for proteins in oligomeric states (that is, for indirect interactions between the sites each in a different subunit)^1^. Although a few studies on the design of allosteric sites have been reported, both the designed allosteric and active sites were in the same monomer^23,24^. We achieved the design of the allosteric site that controls the active site in the other subunit: ATP binding to the designed allosteric site in the B subunit facilitated ADP release at the active site in the A subunit. Remarkably, this designed allostery enabled acceleration of rotation, not deceleration, compared to the wild-type (mutations impairing functions are readily found in general; however no mutations accelerating rotary motors appear to have been reported). In other words, we created cooperativity in the rotary motor. Moreover, the acceleration was tunable by modulating the nucleotide-binding ability of the designed allosteric site. The indirect interactions between the sites each in a different subunit, the accelerated rotation and its tunability indicate that we succeeded in creating an allosteric mechanism in the protein complex, V_1_-ATPase, for controlling the concerted function.

Pseudoenzymes have been reported to account for 5–10% of proteins that make up enzyme families^4^. Hence, pseudo-active sites are widespread in protein complexes, for example, F_1_-ATPase^25^, dynein^6,26^, kinesin^27^, 20S proteasome^28^, kinases^7^ and plant NLR complex^5^. Functional controls of protein complexes can be programmed by various ways^29,30^, but restoring pseudo-active sites could be a promising approach to creating allosteric control for a variety of protein complexes, as we demonstrated. Moreover, beyond their restoration, engineering pseudo-active sites to have novel functions such as binding ability to artificial small molecules will be an interesting challenge.

## Methods

### Statistical analysis of native P-loops

P-loops are found in the connections of successive secondary-structure elements from β-strand to α-helix. Statistical analysis of 52 X-ray structures containing the P-loop (GX_1_X_2_X_3_X_4_G(T/S)), collected from the PISCES server^34^ with resolution ≤2.0 Å, *R* factor ≤0.25 and sequence identity ≤25%, revealed the three conserved features (the orientation, the typical backbone geometry and the amino acid sequence) and the typical distance to the phosphate group of ATP.

### Computational design protocol

The B subunit was computationally redesigned using the structure chain E of V_1_-ATPase (PDB: 3VR6). First, the pseudo P-loop in the B subunit (residues 151–158) was replaced by the P-loop of the A subunit (residues 232–239), by superimposing the A subunit (chain B in the same PDB 3VR6) to the B subunit with the orientation feature of the P-loop shown in Fig. 2, left bottom (1). Second, ATP-binding modes were designed using Rosetta design software^35^ with the Talaris2014 score function (parameters for ATP-Mg^2+^ were determined using those for atom types already defined in Rosetta). In the design calculation, side-chain conformations for the residue positions of the P-loop and the surrounding residues (E169, T248, Q339 and F417), having favourable interactions with ATP, were explored with various ATP conformations generated by BCL software^36^ and with the distance constraints between the atoms of the P-loop and the phosphate atoms of ATP (Supplementary Fig. 2) (the amino acid at X_3_ in the P-loop was fixed to Gly (Fig. 2, left bottom (3))). Third, the designed B-subunit structures with ATP were energetically minimized. The second and third steps were iterated 20 times and 800 different ATP-binding modes were designed. Fourth, the designed structures that lost the feature for conserved backbone torsion pattern of the P-loop (Fig. 2, left bottom (2)) were abandoned, and then those of which ATP-binding score (Rosetta ddG score) are less than −8.0 were finally selected (29 designs).

### Molecular dynamics simulations

The 29 designs obtained by the computational design using Rosetta were further evaluated for their ATP-binding ability by observing the stability of ATP in the designed site during short molecular dynamics simulations. The AMBER14 software suite^37^ was used for all molecular dynamics simulations. The design models were used as the initial structures, of which hydrogen atoms were added by the LEaP module of AMBER14 (ref. ^37^). The simulation system contains a designed B-subunit monomer with ATP placed in a water box of approximately 82 Å × 112 Å × 100 Å. To neutralize the system, 15–17 sodium ions were put in the box. AMBER ff99SB sets and TIP3P were utilized for the protein and water molecules, respectively. Parameters for ATP molecule were adopted from a reference paper^38^. Long-range electrostatic interactions were treated by the particle mesh Ewald method. Non-bonded interactions were cut off at 10 Å. After carrying out a short minimization to remove artificial repulsions in the initial structure, 10 ns molecular dynamics simulations in a constant-NPT (300 K, 1 atm) ensemble were performed after the 100 ps heating stage with the NVT ensemble (the time step is 2.0 fs and hydrogen atoms were constrained with the SHAKE procedure). At the heating step, the temperature was raised gradually from 0 K to 300 K with weak restraints (10 kcal mol^−1^ A^−^^2^) to the atoms of the designed B subunit. The molecular dynamics simulation trajectories for each designed structure are shown in Supplementary Fig. 5 with the r.m.s.d. values for the heavy atoms of the ATP molecule taken from the minimized structure. Finally, a designed structure showing a low r.m.s.d. value throughout the molecular dynamics simulation was selected for experimental characterization.

### Preparation of the design mutants

The mutations were introduced by the Quick Change Multi Site-Directed Mutagenesis Kit (Agilent Technologies). The purification and expression were carried out with the same method as the original design. The DNA sequence was confirmed by DNA sequencing analysis (Fasmac).

### Expression and purification of the A_3_(De)_3_ complex

A DNA fragment of the design was synthesized from the *ntpB* gene in pTR19-AB^21^ using megaprimer PCR method, and then the *ntpB* gene was replaced by this design fragment. The DNA sequence of design plasmid was confirmed by DNA sequencing analysis (Fasmac). *E. coli*. BL21* (DE3) competent cells were transformed with the plasmid and cultured at 30 °C for 20 h in Super Broth (32 g l^−1^ Tryptone, 20 g l^−1^ yeast extract and 5 g l^−1^ sodium chloride) containing 100 μg ml^−1^ ampicillin and 2 mM isopropyl β-d-thiogalactopyranoside. Grown cells were spun down at 6,000 r.p.m. (5,514*g*) for 10 min (Thermo Scientific, Sorvall Legend XTR) and washed twice with buffer A (20 mM potassium Pi (pH 7.0) and 100 mM NaCl). The cells were suspended in 15 ml of buffer A supplemented with 75 μl 100 mM phenylmethylsulfonyl fluoride (PMSF) solution and subsequently disrupted by sonication. After removing cell debris by centrifugation at 10,000 r.p.m. (11,629*g*) for 20 min at 4 °C, the solution was filtered and applied to a Ni-NTA column. After washing with buffer B (20 mM potassium Pi (pH 7.0), 230 mM NaCl and 20 mM imidazole), A_3_(De)_3_ complex was eluted with buffer C (20 mM potassium Pi (pH 7.0), 50 mM NaCl and 250 mM imidazole). The eluted fractions were concentrated with a Vivaspin20 5,000 molecular weight cut-off (Sartorius) and then passed through a Superdex 200 Increase column (GE Healthcare) equilibrated with buffer D (20 mM MES-NaOH (pH 6.5), 100 mM KCl, 5 mM MgSO_4_, 0.1 mM dithiothreitol (DTT) and 10% glycerol). The purified proteins were stored at −80 °C. The methods described above were also used for expression and purification of the wild-type A_3_B_3_ complex.

### Expression and purification of the A_3_(De)_3_ for crystallization

The A_3_(De)_3_ protein sample for crystallization was prepared by cleaving the His-tag attached to the N-terminal part of the A subunit. The TEV protease cleavage site was inserted between the *ntpA* gene and His-tag in pTR19-AB^21^ with a KOD-Plus-Mutagenesis Kit (Toyobo). With this plasmid, the A_3_(De)_3_ sample was expressed and purified by using an Ni-NTA column according to the same protocol described above. The eluted sample and TEV protease were mixed in 10:1 molar ratio and dialysed against buffer J (20 mM Tris–HCl (pH 8.0) and 50 mM NaCl). These dialysed samples were applied to a Ni-NTA column and the flow-through was collected. Then, the sample was loaded onto a HiTrap Q HP column (GE Healthcare Life Sciences) equilibrated with buffer J, and then eluted with a linear gradient of buffer J with 50–1,000 mM NaCl in 20 min at a flow rate of 1.0 ml min^−1^. The concentrated sample with a Vivaspin20 5,000 molecular weight cut-off (Sartorius) was loaded onto a Superdex 200 Increase 10/300 GL column (GE Healthcare) equilibrated with buffer K (20 mM Tris–HCl (pH 8.0), 150 mM NaCl and 2 mM DTT) at a flow rate of 0.5 ml min^−^^1^. The purified samples were concentrated with a Vivaspin500 5,000 molecular weight cut-off.

### Crystallization, data collection and structure determination

The sitting drop vapour diffusion method was used for crystallization. Crystals for A_3_(De)_3__empty were obtained by mixing a 2.0 μl protein solution drop (10–15 mg ml^−1^ protein in buffer K) with 2.0 μl of reservoir solution (0.1 M Tris–HCl (pH 8.5), 20–24% PEG 3350 and 0.2 M ammonium acetate). The crystals appeared in 1–2 weeks at 293 K. The crystals were soaked in cryoprotectant solutions with an increasing concentration of 10% (v/v) glycerol. For A_3_(De)_3__(ADP·Pi)_1cat_(ADP)_2cat,2non-cat_, A_3_(De)_3__empty crystals were soaked in ADP, MgCl_2_ and glycerol for 5 h by gradually increasing the concentration to 10 mM, 10 mM and 10%, respectively. For A_3_(De)_3__(ADP)_3cat,1non-cat_ and A_3_(De)_3__(ADP)_3cat,2non-cat_, A_3_(De)_3__empty crystals were soaked in ADP, MgCl_2_ and glycerol overnight by gradually increasing the concentration to 5 mM, 5 mM and 10%, respectively.

The crystals were mounted on cryo-loops (Hampton Research), flash-cooled and stored in liquid nitrogen. All X-ray diffraction data were collected at a wavelength of 1.1 Å on beamline BL-1A at Photon Factory (Tsukuba, Japan), from a single crystal at the cryogenic temperature (100 K). The collected data were processed using XDS^39^. The structures of A_3_(De)_3__empty and A_3_(De)_3_ with nucleotides were determined by the molecular replacement method with Phaser^40^ using the A_3_B_3_ complex from *E. hirae* (PDB 3VR2) and the obtained A_3_(De)_3__empty structure as a search model, respectively. The initial model was iteratively refined with PHENIX^41^ and REFMAC5(CCP4 Suite)^42^ and manually corrected with COOT^43^. Figures were prepared by PyMOL^44^, CueMol2 (ref. ^45^) and Chimera^46^. All F_o_ − F_c_ omit maps were calculated by phenix.polder^47^ in PHENIX. When densities corresponding to the position of pyrophosphate and ribose were observed, we considered that ADP binds to nucleotide-binding sites. The crystallographic and refinement statistics are summarized in Supplementary Table 5.

### Expression and purification of the designed B-subunit monomer

The designed B-subunit monomer was obtained by breaking the A_3_(De)_3_ complex sample in the presence of a high concentration ATP. After expression and Ni-NTA purification of the A_3_(De)_3_ complex sample by the methods described above, the buffer of the A_3_(De)_3_ sample solution eluted from a Ni-NTA column was exchanged to buffer M (20 mM MES-NaOH (pH 6.5), 10% glycerol, 100 mM KCl and 5 mM MgSO_4_) using a PD10 column (GE Healthcare). The sample solution mixed with 2 mM ATP was rocked for 30–40 min at 4 °C, filtered and applied to a Ni-NTA column. Because the A subunit has a His-tag and the designed B subunit does not, the designed B subunit can be selectively recovered in the flow-through. In the flow-through sample, Tris–HCl was added (100 mM final concentration, pH 8.5). The buffer of sample solution was exchanged to buffer N (20 mM Tris–HCl (pH 8.5), 10% glycerol, 100 mM KCl and 5 mM MgSO_4_) by concentrating with a Vivaspin20 5,000 molecular weight cut-off (Sartorius) and adding buffer N. The samples were passed through a Superdex 200 Increase column (GE Healthcare) equilibrated with buffer N. The purity of designed monomer sample was confirmed by SDS-PAGE (Supplementary Fig. 7c).

### Expression and purification of the wild-type B-subunit monomer

The wild-type B-subunit monomer was expressed with pTR19-B plasmid, which is constructed from the pTR19-AB plasmid^21^ by deleting the *ntpA* gene and adding His-tag to the *ntpB* gene, using the same protocol used for the A_3_(De)_3_ complex. The cells were suspended in 25 ml of buffer O (20 mM Tris–HCl (pH 8.5), 5% glycerol, 0.7 M KCl, 5 mM MgSO_4_, 0.1 mM DTT and 20 mM imidazole (pH8.5)) supplemented with 125 μl of 100 mM PMSF solution, and then disrupted by sonication. After removing cell debris by centrifugation at 10,000 r.p.m. (11,629*g*) for 20 min at 4 °C (Thermo Scientific, Sorvall Legend XTR), the solution was filtered and applied to an Ni-NTA column. After washing with buffer P (20 mM Tris–HCl (pH 8.5), 5% glycerol, 0.7 M KCl, 5 mM MgSO_4_, 0.1 mM DTT and 20 mM imidazole (pH8.5)), B-subunit monomer was eluted with buffer Q (20 mM Tris–HCl (pH 8.5), 5% glycerol, 0.7 M KCl, 5 mM MgSO_4_, 0.1 mM DTT and 250 mM imidazole (pH8.5)). The eluted fractions were concentrated with a Vivaspin20 5,000 molecular weight cut-off (Sartorius) and then passed through a Superdex 200 Increase column (GE Healthcare) equilibrated with buffer N. The purified proteins were stored at −80 °C.

### Circular dichroism measurement

Thermal denaturation experiments for the designed and the wild-type B-subunit monomers were carried out using a circular dichroism spectrometer (J-1500KS, JASCO). Far-ultraviolet circular dichroism spectra at 220 nm with temperature increasing in steps of 1.0 °C min^−1^ with 60 s of equilibration time were collected for 5 μM protein samples in buffer N in a 1-cm-path-length cuvette. The pH of the buffer was adjusted to 8.5 after the addition of nucleotides. The measurements were carried out three times independently in the absence of nucleotides or in the presence of 0.5, 1.0, 2.0 mM [ADP] or [ATP] after the incubation of the mixed solutions for 1 h at 4 °C.

### ATPase activity measurements for monomers

ATPase activities for the wild-type A- and B-subunit monomers and the designed B-subunit monomer were measured with an ATPase/GTPase activity assay kit (MAK113, Sigma-Aldrich). Proteins (250 nM) were incubated with 1 mM ATP in 40 μl assay buffer (40 mM Tris, 80 mM NaCl, 8 mM MgAc_2_, 1 mM EDTA, pH 7.5) in 96-well plates (Greiner, 655801) for 1 h at room temperature. Solutions without protein samples were incubated as the reference at the same condition. After the incubation, 200 μl malachite green reagents were added to each well and the solutions were incubated for 30 min at room temperature. For the incubated solutions, absorbances at 350–850 nm were measured with a Spark 10M (TECAN) microplate reader. Product (phosphate) concentrations were estimated from the relative absorbance (620 nm) for the reference by comparing with the absorbances for standard buffers at several phosphate concentrations. ATPase activity for the A subunit was calculated from the estimated product concentrations.

### Expression and purification of the DF subcomplex

The DF subcomplex of *E. hirae* V_1_ was expressed in *E. coli*. BL21* (DE3) competent cells using the pTR19-D(M1G/T60C/R131C)F plasmid^21^. The transformed cells were cultured in Super Broth containing 100 μg ml^−1^ ampicillin at 37 °C for 4–5 h until the absorbance at 600 nm reached 0.5; then the temperature was decreased to 30 °C and expression of the DF subcomplex was induced by the addition of 2 mM isopropyl β-d-thiogalactopyranoside. Cells were harvested 20 h after induction by centrifugation at 6,000 r.p.m. (5,514*g*) for 10 min (Thermo Scientific, Sorvall Legend XTR). The cells were suspended in 20 ml of buffer E (20 mM potassium P_i_ (pH 8.0), 300 mM NaCl and 20 mM imidazole) supplemented with 100 μl of 100 mM PMSF solution, and then disrupted by sonication. After removal of cell debris by centrifugation at 10,000 r.p.m. (11,629*g*) for 20 min at 4 °C, the solution was filtered and applied to an Ni-NTA column. After washing with buffer E, the DF subcomplex was eluted with buffer F (20 mM potassium P_i_ (pH 8.0), 300 mM NaCl and 500 mM imidazole). The eluted sample and TEV protease were mixed in 10:1 molar ratio and dialysed against buffer G (20 mM potassium P_i_ (pH 8.0), 50 mM NaCl and 1 mM DTT) overnight. The dialysed sample was spun down at 10,000 r.p.m. (11,629*g*) for 20 min at 4 °C, and then applied to a PD10 column for changing to the buffer E. The eluted sample was applied to an Ni-NTA column and the flow-through was collected. After adding 1 mM DTT, the sample was concentrated with a Vivaspin20 5,000 molecular weight cut-off (Sartorius) and then passed through a Superdex 75 column (GE Healthcare) equilibrated with buffer H (20 mM Tris–HCl (pH 8.0), 150 mM NaCl). The purified proteins were stored at −80 °C. For single-molecule experiments, the cysteine residues introduced in the D subunit by the mutations T60C and R131C were biotinylated using the purified DF-subcomplex sample. The buffer of sample solution was changed to buffer I (20 mM potassium P_i_ (pH 7.0), 150 mM NaCl) using a PD10 column. The biotinylation regent (biotin–PEAC_5_–maleimide, Dojindo) was mixed into the purified DF-subcomplex sample solution at a 3:1 molar ratio, and then incubated for 30 min at room temperature. Finally, DTT (10 mM final concentration) was added to the sample solution and the sample was stored at −80 °C. The purification results for gel filtration and SDS-PAGE are shown in Supplementary Fig. 7d.

### Single-molecule experiments of the designed V_1_-ATPase

The protein sample was prepared by mixing the purified A_3_(De)_3_ and the biotinylated DF subcomplex in a 1:5 molar ratio with the addition of MES-NaOH (pH 6.0, 100 mM final concentration), followed by the incubation in the presence of 200 μM ADP for 2 h at room temperature. The sample was filtered and passed through a Superdex 200 Increase column (GE Healthcare) equilibrated with buffer L and concentrated to a few μM with a Vivaspin500 5,000 molecular weight cut-off. The samples were stored at −80 °C. Protein sample preparations were carried out a few times. Almost all data at each ATP concentration were collected using the samples prepared at the same times.

Single-molecule experiments and data analyses were carried out by the method reported in a previous paper^21^. All data for the wild-type are from the previous paper^21^. The flow cell was prepared by covering an untreated coverglass (18 × 18 mm^2^, Matsunami Glass) on a coverglass (24 × 32 mm^2^, Matsunami Glass) treated by overnight immersion in piranha solution (H_2_SO_4_/H_2_O_2_ = 3:1). After capturing the protein sample on the treated coverglass by His-tag, the streptavidin-coated 40 nm gold nanoparticle was attached to the biotinylated DF. For almost all measurements, a flow cell was independently prepared for each measurement. The rotation of the gold nanoparticle was observed by using an objective-type total internal reflection dark-field microscope^48^ constructed on an inverted microscope (IX-70, Olympus). The gold nanoparticles were illuminated by an evanescent field with a penetration depth of 100 nm from the glass surface. The scattered image of a rotating gold nanoparticle was recorded as a movie with a high-speed CMOS camera (FASTCAM 1024PCI, Photron) at 10,000 frames per second for almost all samples and at 27,000 frames per second for dwell-time analyses of the double mutant at 3 mM ATP. During observation and recording under the microscope, an ATP-regeneration system, in which ADP is rapidly regenerated to ATP by the coupling with dephosphorylation of phosphoenolpyruvate catalysed by pyruvate kinase, was used to keep [ATP] constant. All fittings against dwell time and angle distributions were carried out by least-squares fitting.

### ATPase activity measurements of V_1_ complexes

ATPase activity measurements in solution were conducted in a similar way to the previous research^49^. ATPase activities for the wild-type and designed V_1_-ATPase were measured at 25 °C in 50 mM MES-KOH (pH 6.5) containing 50 mM KCl, various concentrations of MgCl_2_ and ATP, and the ATP-regenerating system supplemented with 0.2 mM NADH, 0.1 mg ml^−1^ pyruvate kinase and 0.1 mg ml^−1^ lactate dehydrogenase. ATPase activities were calculated from the slope obtained from the absorbance data of NADH during 20 s after the start of measurement. The measurements were performed three times independently at each [ATP].

### Reporting summary

Further information on research design is available in the Nature Portfolio Reporting Summary linked to this article.

## Online content

Any methods, additional references, Nature Portfolio reporting summaries, source data, extended data, supplementary information, acknowledgements, peer review information; details of author contributions and competing interests; and statements of data and code availability are available at 10.1038/s41557-023-01256-4.

### Supplementary information

Supplementary Information Supplementary Figs. 1–17, Tables 1–5, Text and References.

Reporting Summary

Supplementary Data 1 Computational design model.

Supplementary Data 2 Initial coordinate of MD simulation.

Supplementary Data 3 Final coordinate of MD simulation.

Supplementary Data 4 Design sequences and primers and raw data for supplementary figures.

### Source data

Source Data Fig. 4 Raw data of rotation from single-molecule experiments.

Source Data Fig. 5 Raw data of rotation from single-molecule experiments and raw data of the analysed time constants.

## Data Availability

The data supporting the findings of this work are available in the main and extended figures and the Supplementary Information. The crystal structures have been deposited in the wwPDB as PDB 8IGU (A_3_(De)_3__empty), 8IGV (A_3_(De)_3__(ADP·Pi)_1cat_(ADP)_2cat,2non-cat_) and 8IGW (A_3_(De)_3__(ADP)_3cat,1non-cat_ and A_3_(De)_3__(ADP)_3cat,2non-cat_). The plasmid encoding the designed V_1_-ATPase is available from the authors upon request. The designed model structure, the initial and final coordinates of the molecular dynamics simulation and Source data are provided with this paper.
