# Supplementary material for: Design of allosteric sites into rotary motor V1-ATPase by restoring lost function of pseudo-active sites
Source: Nat Chem. 2023 Jul 6;15(11):1591–8. doi: 10.1038/s41557-023-01256-4 (PMC10624635; doi:10.1038/s41557-023-01256-4)
Supplement: Supplementary file 1 — Supplementary Figs. 1–17, Tables 1–5, Text and References. [file 41557_2023_1256_MOESM1_ESM.pdf]

nature chemistry

Article

https://doi.org/10.1038/s41557-023-01256-4

Design of allosteric sites into rotary motor V_1-ATPase by restoring lost function of pseudo-active sites

In the format provided by the authors and unedited

Supplementary Figures

Supplementary Fig. 1: Structural similarities between A- and B-subunits

Supplementary Fig. 2: Typical binding atom-pair distances with phosphate group of ATP

Supplementary Fig. 3: Design target residues for engineering the B-subunit's pseudo-active site

Supplementary Fig. 4: Purification of the $A_3(De)_3$ complex, in which many residue positions in the B-subunit were used for the design

Supplementary Fig. 5: ATP binding abilities of the 29 designs, evaluated by short MD simulations

Supplementary Fig. 6: Flowchart for designing ATP binding site in the B-subunit's pseudo-active site

Supplementary Fig. 7: Gel filtration chromatography and SDS-PAGE of the designed complexes, the designed B-subunit monomer and the DF-subcomplex

Supplementary Fig. 8: Stereo view of Fo-Fc omit map for ADP and Mg^{2+} molecules

Supplementary Fig. 9: Thermal shifts of the wild-type and designed B-subunit monomers upon nucleotide binding

Supplementary Fig. 10: ATPase activity assay for the wild-type A- and B-subunit monomer and the designed B-subunit monomer

Supplementary Fig. 11: The fitting of the rotation rate data for the designed V_1 to the Michaelis-Menten equation

Supplementary Fig. 12: Comparisons for the rotation rates between the wild-type and designed V_1 at several [ATP]s

Supplementary Fig. 13: ATPase activities of the wild-type and designed V_1 in solution at several [ATP]s

Supplementary Fig. 14: Duration time distributions with estimated time constants for main- and sub-pauses and $\Delta\theta$ for the designed V_1 and the design double mutant K157A/S158A

Supplementary Fig. 15: Duration time distributions with estimated time constants for main- and sub-pauses and $\Delta\theta$ for individual molecules

Supplementary Fig. 16: Comparisons for the sub-pause time constants between the wild-type and designed V_1 at several [ATP]s

Supplementary Fig. 17: Structural comparison between the catalytic site in the presence and absence of an ADP molecule at the neighboring designed site

Supplementary Tables

Supplementary Table 1: Number of molecules used for measurement of average rotation rates in the single-molecule experiments

Supplementary Table 2: Rosetta ddG score calculations to evaluate ATP binding ability of the designed V_1 and its mutants

Supplementary Table 3: Comparison of rotation rates estimated from the slope in the time course of rotation and those estimated from the dwell time constants for the main- and sub-pauses

Supplementary Table 4: Structural comparison of the catalytic interfaces between the nucleotide-free A_3B_3 complex of the wild-type (3VR2) and designed V_1 ($A_3(De)_3$_$(ADP)_{3cat,1non\text{-}cat}$ and $A_3(De)_3$_$(ADP)_{3cat,2non\text{-}cat}$)

Supplementary Table 5: Data collection and refinement statistics of crystal structures

Supplementary Text

1. Designed B-subunit monomer binds to nucleotide in solution
2. Rosetta scripts XML file for designing ATP binding site

Supplementary Data Figures

Uncropped scans of gels in Supplementary Fig. 4 and 7

Supplementary References

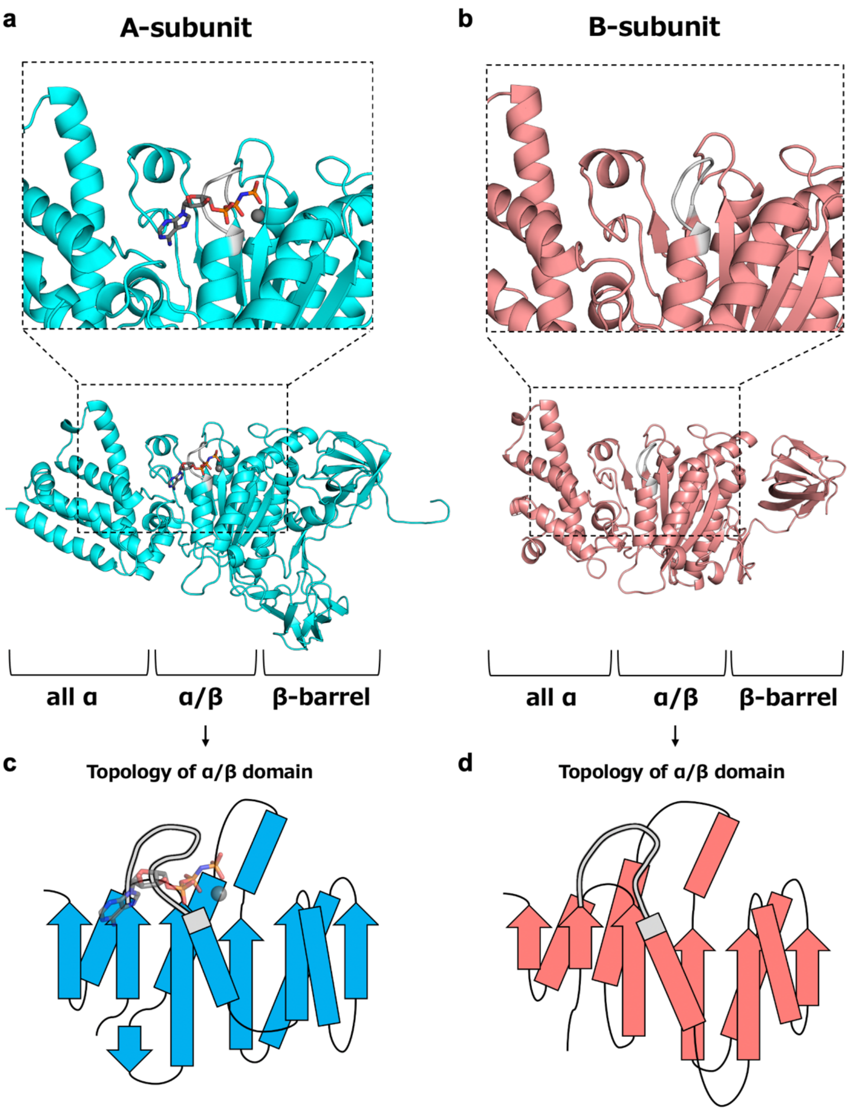

Supplementary Fig. 1: Structural similarities between A- and B-subunits. The A- and B-subunit are presented at left and right, respectively. **a and b**, Overall structures of the A- and B- subunits with close-up views of structures around the active site in the A-subunit and the pseudo-active site in the B-subunit. Both subunits consist of all-α-, α/β-, and β-barrel domains, and have similar backbone structure (TM-score evaluated by MICAN[1] is 0.87). The spatial arrangement of secondary structures around the pseudo-active site in the B-subunit is nearly the same as those of the active site in the A-subunit. **c and d**, Topology schematics of α/β domains of the A- and B- subunits. The α/β domain of the B-subunit (residue 78-362) has an almost identical topology with that of the A-subunit (residue 72-449).

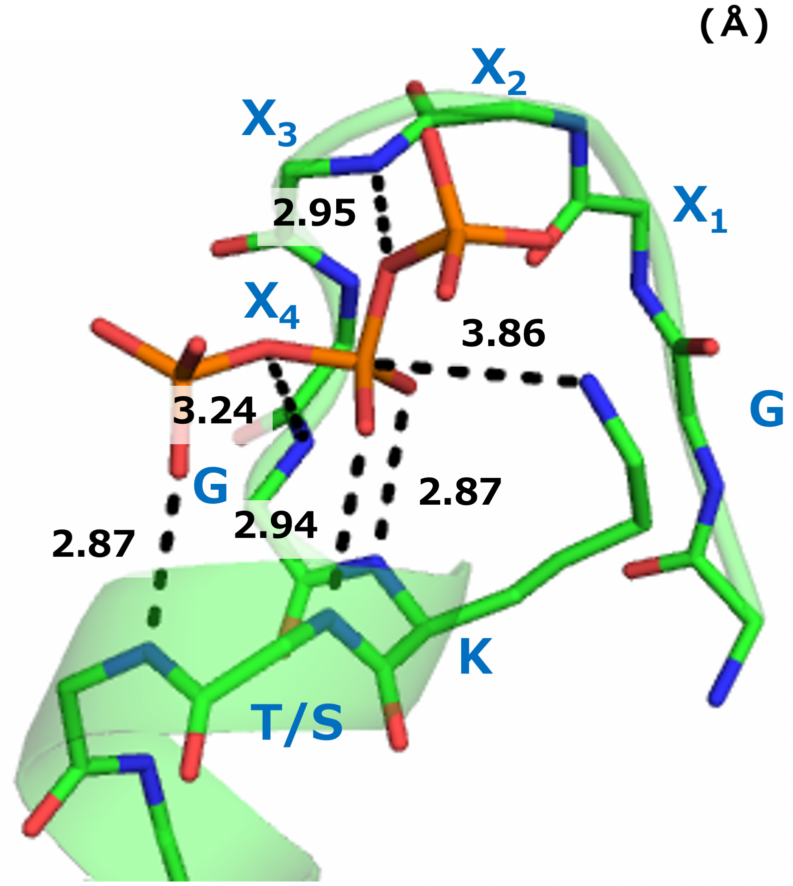

Supplementary Fig. 2: Typical binding atom-pair distances with phosphate group of ATP. The typical distances between the atoms of P-loop and the phosphate atoms of ATP were used for engineering ATP-binding ability of the B-subunit's pseudo-active site. Each distance was obtained by averaging the corresponding distances of the P-loop motifs, which we collected from naturally occurring proteins in this work.

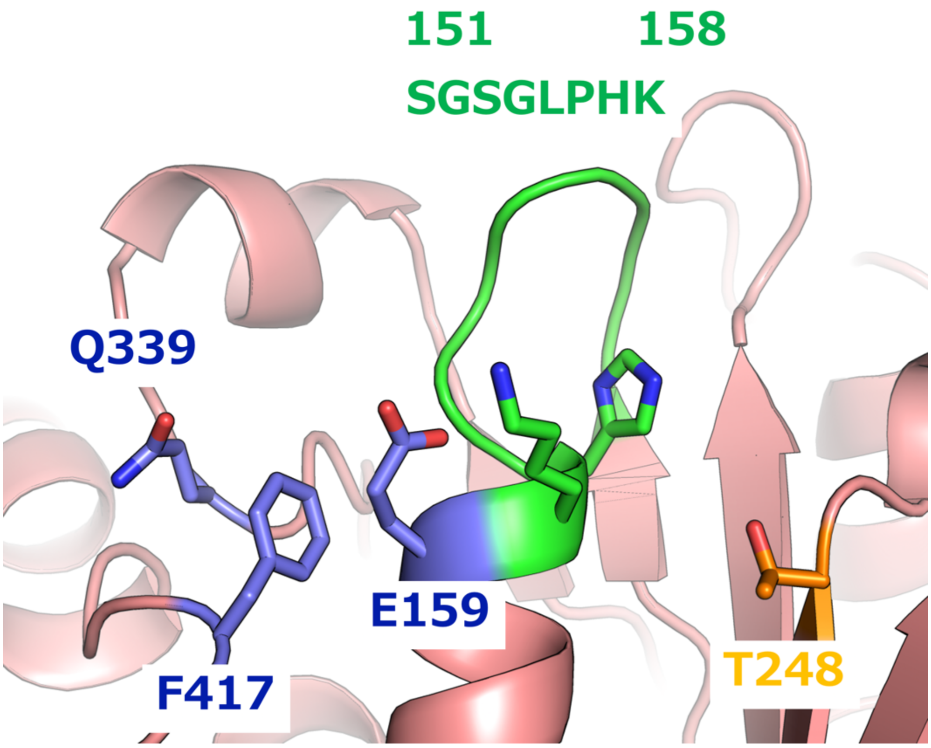

Supplementary Fig. 3: Design target residues for engineering the B-subunit's pseudo-active site. Residue positions in the pseudo P-loop from 151 to 158 (green) were designed as a P-loop motif ($GX_1X_2X_3X_4G$[T/S]). The amino acid type at X_3 position in the P-loop motif was fixed to Gly because of the high conservation (Fig. 2 left bottom (3)). T248 (orange) was designed using Asp or Glu to introduce the Walker B motif. The three purple residues were selected to provide a space for nucleotide binding. The number of target residues was kept as low as possible, because the V_1 lost the ability to form the complex when more residues were selected for the design (Supplementary Fig. 4).

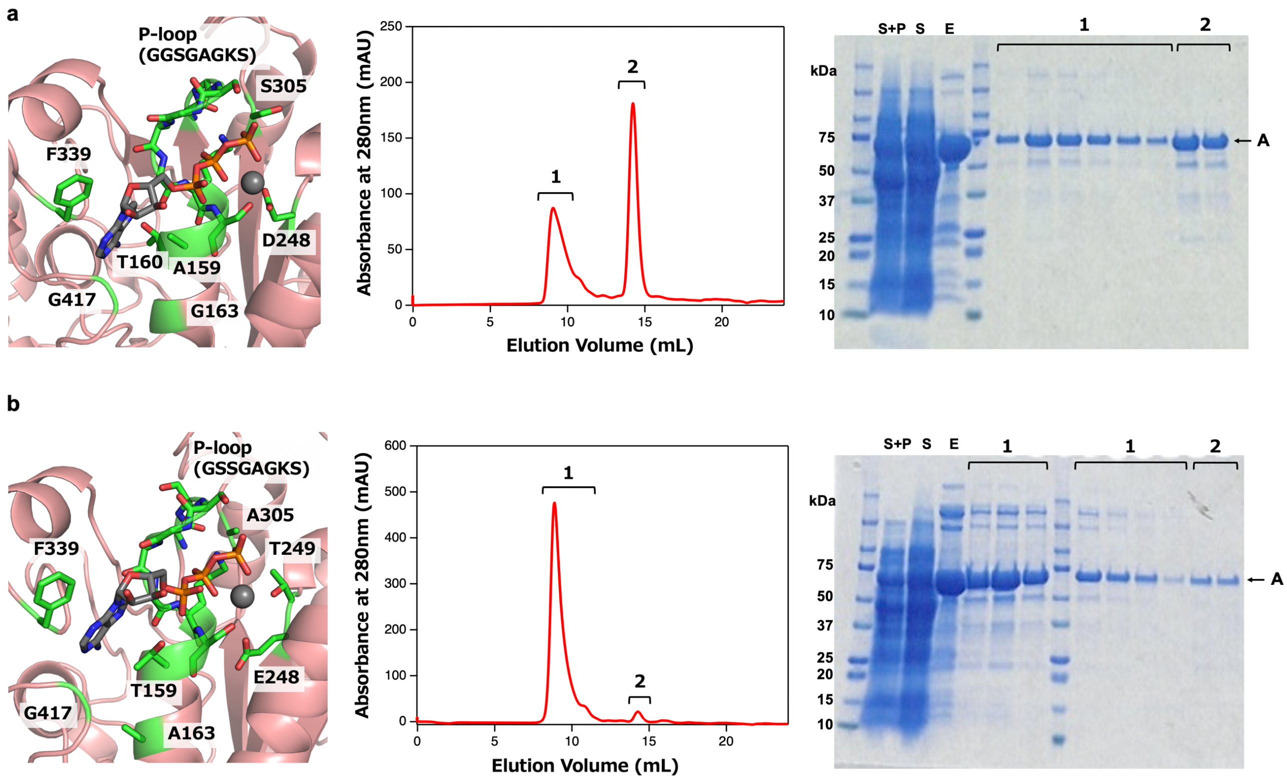

Supplementary Fig. 4: Purification of the $A_3(De)_3$ complex, in which many residue positions in the B-subunit were used for the design. Left: Residue positions used for the design: 18 positions for **a** and 21 positions for **b**. Middle and Right: purification results for gel filtration chromatography and SDS-PAGE (S+P, S and E indicate supernatant+pellet, supernatant, and elution, respectively). For the purification of the $A_3(De)_3$ complex, only the A-subunits have a His-tag, but the B-subunits do not. For both designs, only the A-subunit was identified in SDS-PAGE, indicating that the designed B-subunit was not capable of forming the complex with the A-subunit. Sample expressions and purifications for each design were replicated twice.

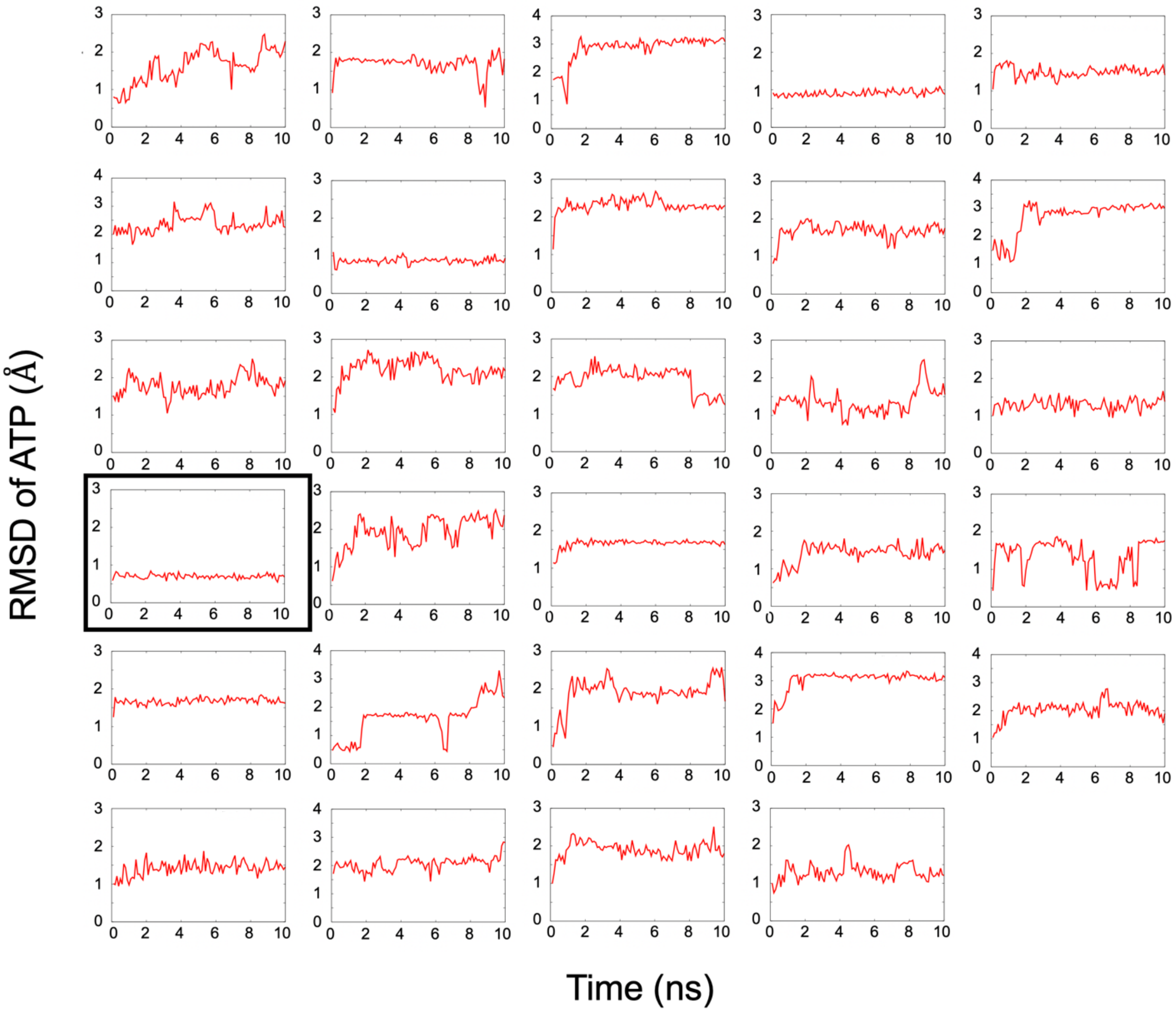

Supplementary Fig. 5: ATP binding abilities of the 29 designs, evaluated by short MD simulations. In each panel, RMSD values calculated using heavy atoms of ATP molecule in a designed structure are plotted along the time course. The MD trajectory surrounded by the thick line frame is the design selected for experimental characterizations.

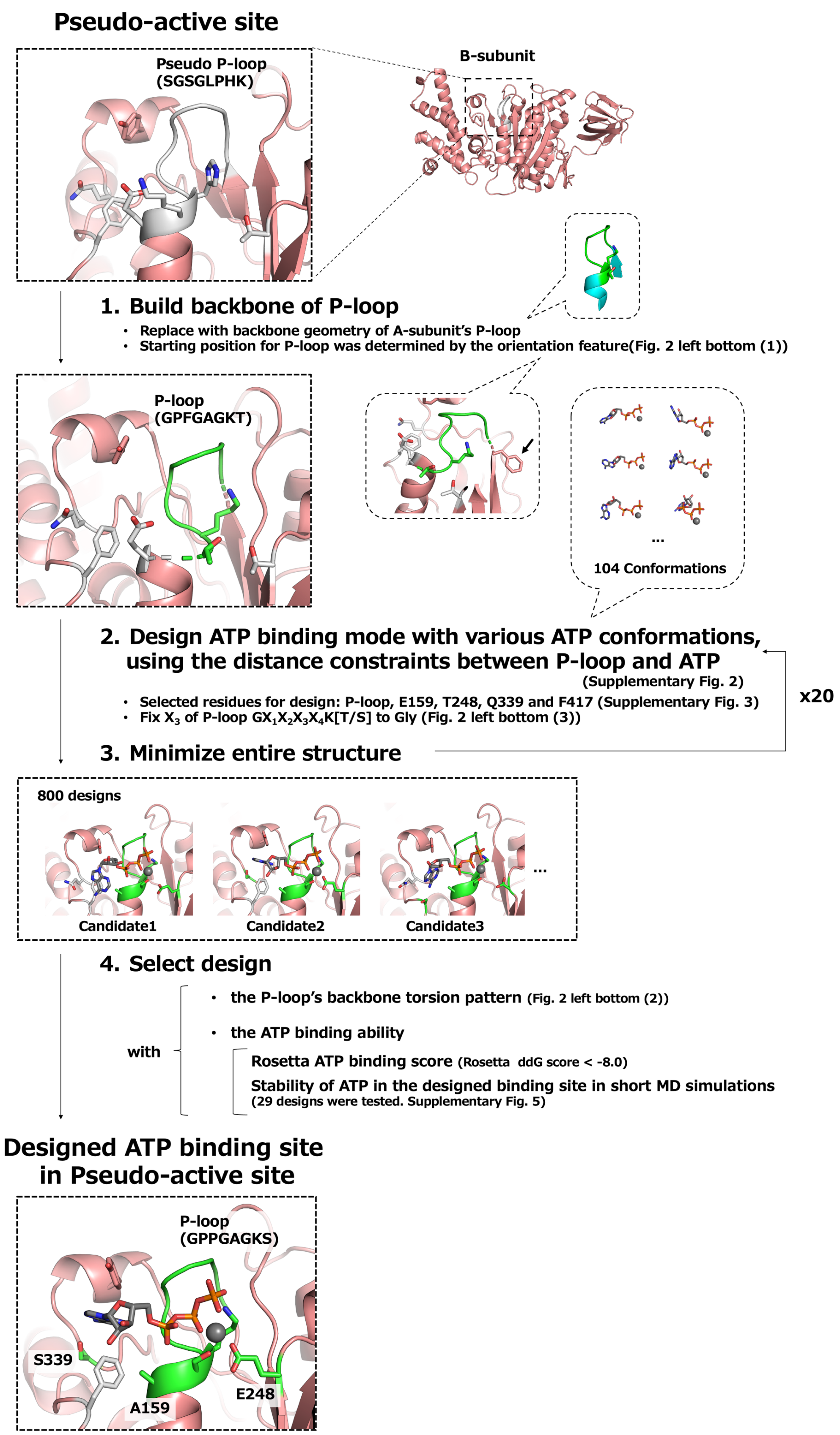

Supplementary Fig. 6: Flowchart for designing ATP binding site in the B-subunit's pseudo-active site.

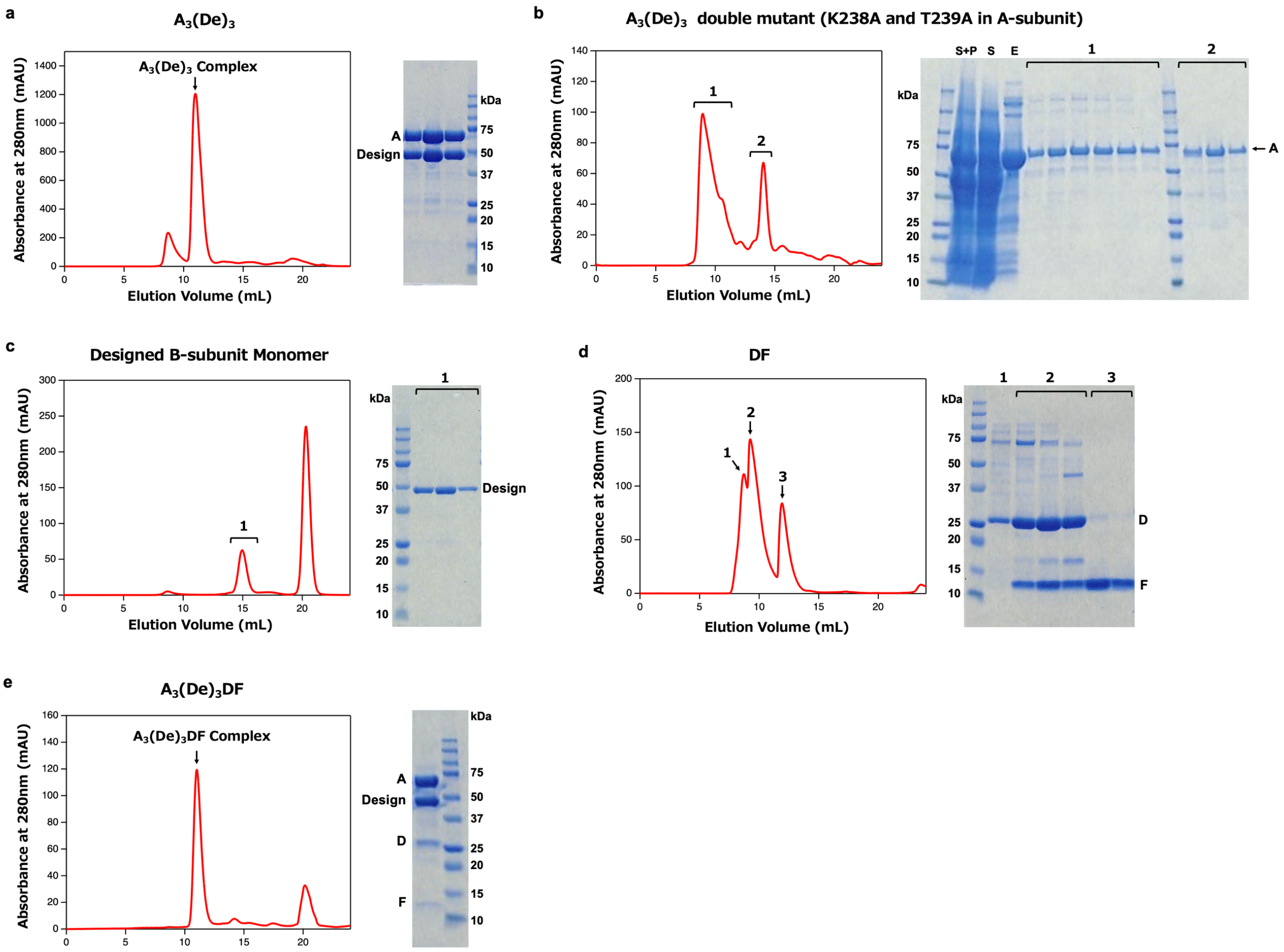

Supplementary Fig. 7: Gel filtration chromatography and SDS-PAGE of the designed complexes, the designed B-subunit monomer and the DF-subcomplex. a, The $A_3(De)_3$ complex. The $A_3(De)_3$ was purified as the complex. **b,** The $A_3(De)_3$ with the double mutant K238A/T239A in the A-subunit. The A-subunit double mutant K238A/T239A did not form the complex with the designed B-subunit. **c,** The designed B-subunit monomer. The $A_3(De)_3$ complex was expressed and purified, and subsequently broken by adding an excessive amount of ATP. The designed B-subunit monomer was then collected from the peak of gel filtration. **d,** The DF-subcomplex. The DF-subcomplex was purified as the complex. **e,** The $A_3(De)_3DF$ complex. The $A_3(De)_3$ complex was mixed with the DF-subcomplex. In SDS-PAGE, S+P, S and E indicate supernatant+pellet, supernatant, and elution, respectively. These sample expressions and purifications were replicated at least twice.

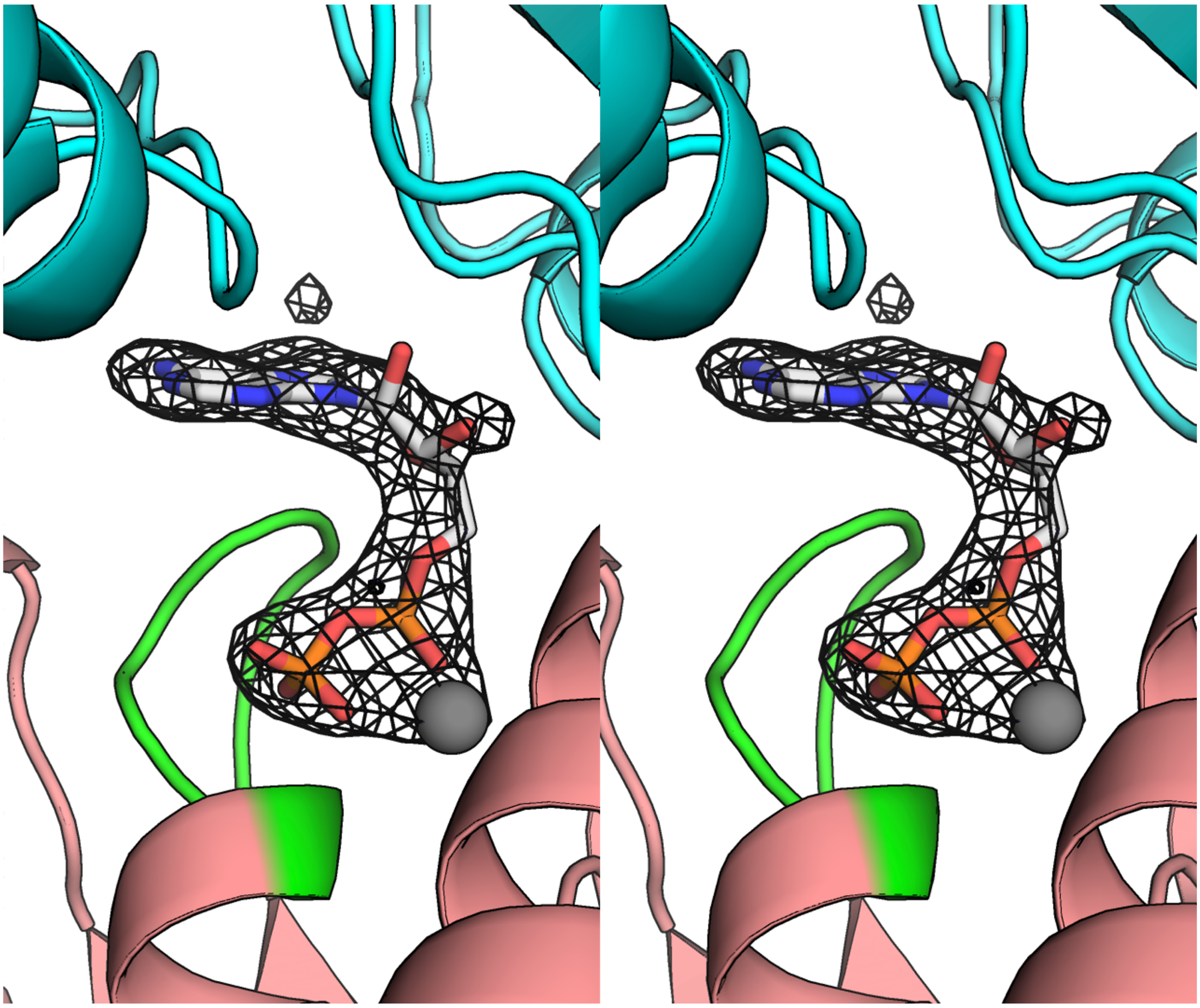

Supplementary Fig. 8: Stereo view of Fo-Fc omit map for ADP and Mg^{2+} molecules. The interface between the chain C (cyan) and D (pink) in $A_3(De)_3$_$(ADP{\cdot}Pi)_{1cat}(ADP)_{2cat,2non\text{-}cat}$ is shown. The designed P-loop (green) and F_o-F_c omit map at 3.0σ for ADP and Mg^{2+} molecules (mesh) are shown.

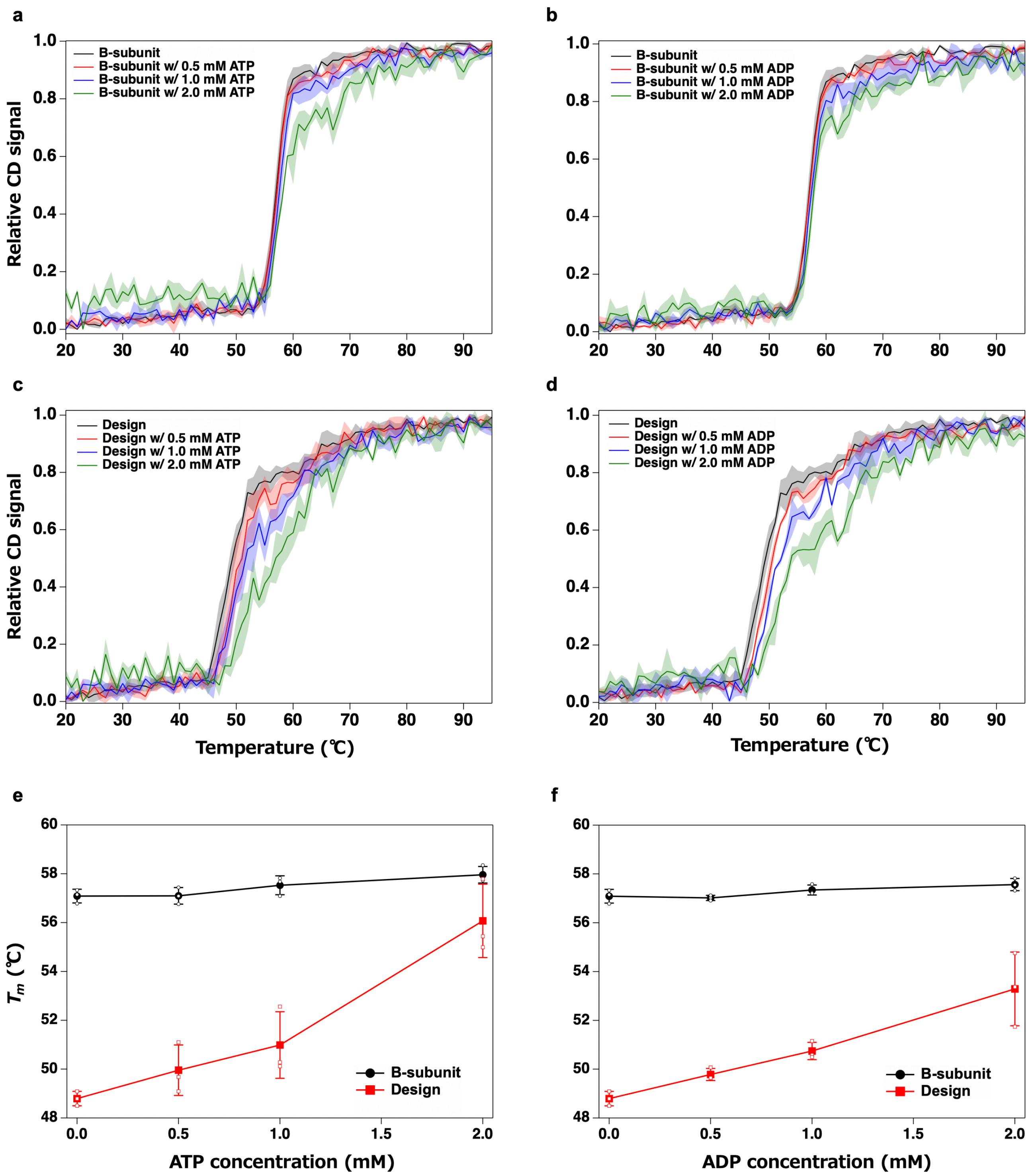

Supplementary Fig. 9: Thermal shifts of the wild-type and designed B-subunit monomers upon nucleotide binding. Thermal denaturation curves for the wild-type B-subunit monomer (**a and b**) and the designed B-subunit monomer (**c and d**) in the presence and absence of ADP or ATP, measured by circular dichroism (CD) at 220 nm, are shown. The CD values in the denaturation curves were

normalized between 0 and 1 by min-max normalization: (CD signal – the lowest CD signal)/(the highest CD signal – the lowest CD signal)). The error bars represent S.D. **e and f,** Melting temperature (T_m) values at different [ATP]s or [ADP]s are shown. While the wild-type B-subunit shows almost the same T_m values both in the presence and absence of nucleotide, the design monomer exhibits higher T_m values in the presence of ATP or ADP than those in the absence of nucleotide. These measurements were carried out individually three times and the average values were plotted with the error bars representing S.D. The T_m values for the wild-type and designed B-subunits were obtained from the denaturation curves by non-linear least-squares analysis using a two-state unfolding and linear extrapolation model.

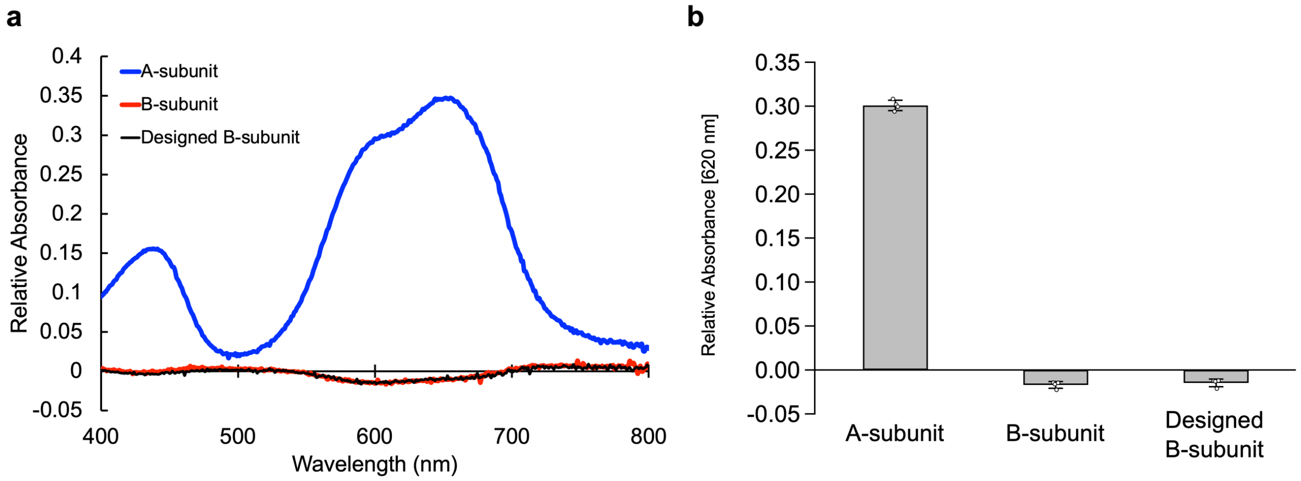

Supplementary Fig. 10: ATPase activity assay for the wild-type A- and B-subunit monomer and the designed B-subunit monomer. a, Relative absorbances, which were calculated by subtracting the absorbance for ATP solution incubated without protein samples, for the A-subunit (blue), the B-subunit (red) and the designed B-subunit (black). **b**, Averaged relative absorbances at 620 nm for the A-subunit, the B-subunit and the designed B-subunit. The averaged absorbances and standard errors were calculated for four independent experiments. The estimated ATPase activity of the A-subunit was 2.15 ± 0.04 ATP molecules per minute. Note that the ATPase activity of the single A-subunit in the wild-type V_1-ATPase complex is approximately 100-120 ATP molecules per second, which is evaluated by single-molecule experiments. ATPase activities of the wild-type and designed B-subunit were not detected.

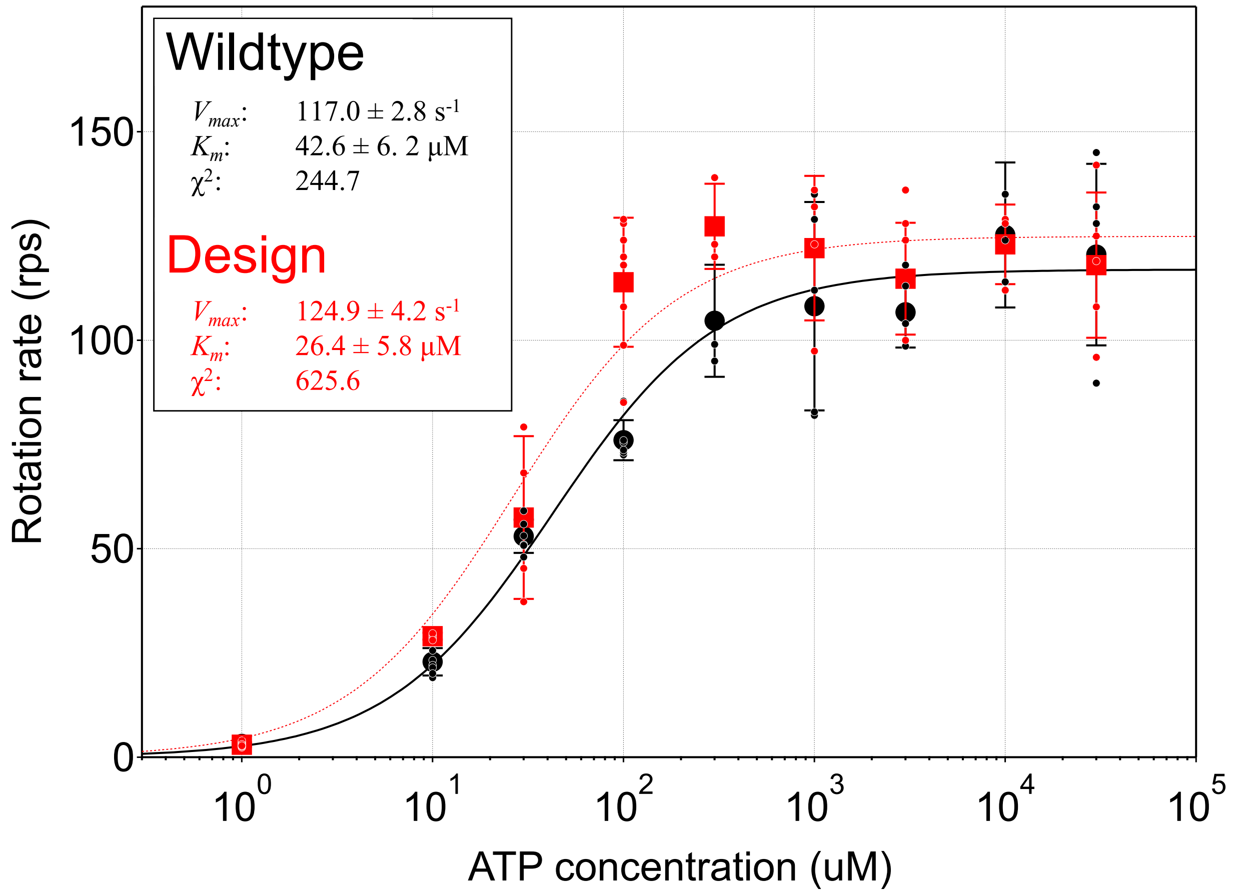

Supplementary Fig. 11: The fitting of the rotation rate data for the designed V_1 to the Michaelis-Menten equation. The rates of the design were plotted with averaged values using three molecules or more (Supplementary Table 1) and the error bars representing S. D. All data for the wildtype are from the previous paper[2]. The black curve is obtained by fitting the rotation rate data of the wild-type V_1 to the Michaelis-Menten equation (V_{max}: 117.0 ± 2.8 s^{-1}, K_m: 42.6 ± 6.2 µM, χ^2: 244.7). The red curve was obtained by fitting the rotation rate data of the designed V_1 to the Michaelis-Menten equation (V_{max}: 124.9 ± 4.2 s^{-1}, K_m: 26.4 ± 5.8 µM, χ^2: 625.6). The data were fitted with the least-squares fitting method. In the fitting, the designed V_1 shows a larger fitting error, χ^2 values, due to the acceleration at a certain [ATP] range.

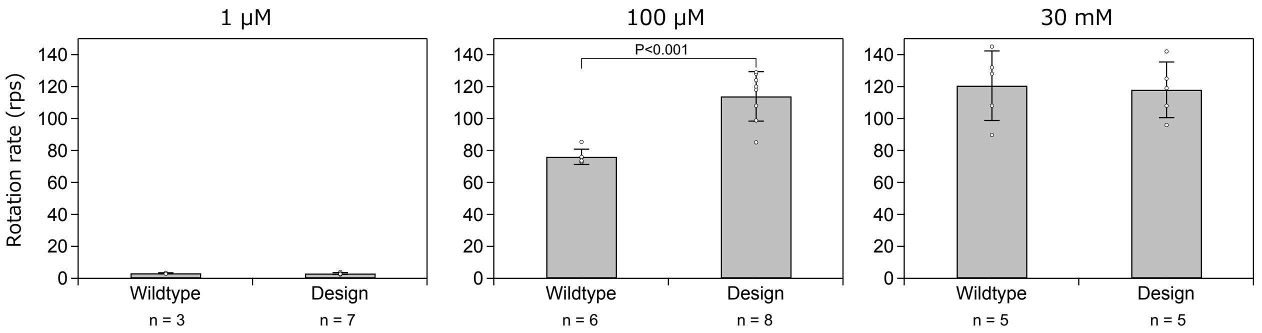

Supplementary Fig. 12: Comparisons for the rotation rates between the wild-type and designed V_1 at several [ATP]s. The rotation rates measured by single-molecule experiments are plotted with averaged values using three molecules or more (n indicates number of analyzed molecules) and the error bars representing S.D. By Welch's two-tailed t-tests for this results, we found a significant difference between the wild-type and designed V_1 at 100 μM ATP (95% confidence interval, $t(9)=6.513$, $p=0.0001$), but did not found at 1 μM and 30 mM ATP. The rate is significantly accelerated at certain range of [ATP]s.

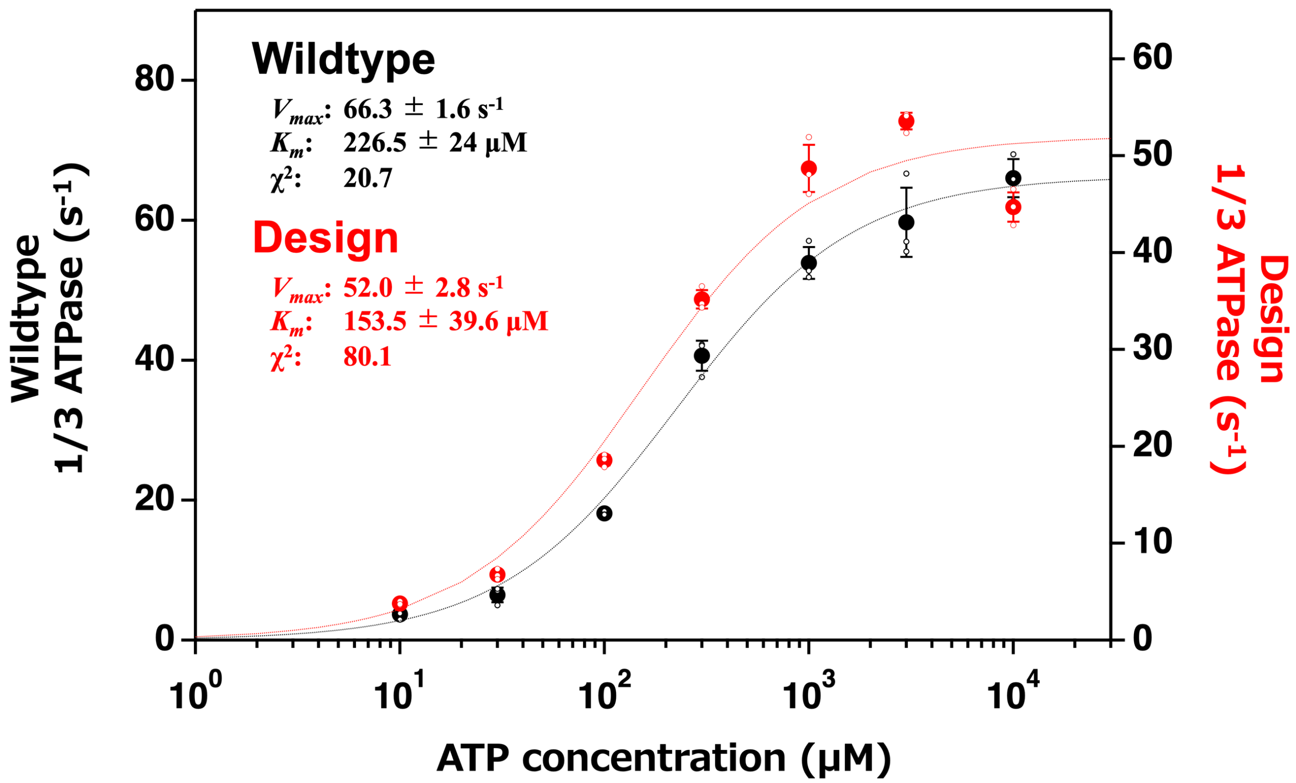

Supplementary Fig. 13: ATPase activities of the wild-type and designed V_1 in solution at several [ATP]s. ATPase activities of the wild-type V_1 (black) and the designed V_1 (red) were measured in solution with the ATP-regeneration system. The activities were plotted with averaged values for three independent experiments and the error bars representing S. D. The [ATP] dependence of the wildtype follows the Michaelis-Menten equation (χ^2 value is 20.7.), while the designed V_1 shows a larger fitting error in the fitting using the Michaelis-Menten equation (χ^2 value is 80.1.). Note that the V_{max} (wildtype: 66.3 ± 1.6 s^{-1}, design: 52.0 ± 2.8 s^{-1}) and K_m (wildtype: 226.5 ± 24 μM, design: 153.5 ± 39.6 μM) values are respectively lower and higher than those obtained by the single-molecule experiments (V_{max} values of the wildtype and design are 117.0 ± 2.8 and 124.9 ± 4.2 s^{-1}. K_m values of the wildtype and design are 42.6 ± 6.2 and 26.4 ± 5.8 μM). This trend has been reported in other researches[3,4]. The reason is that only the molecules that form the intact complex and rotate smoothly are used in single-molecule experiments, while all molecules in solutions are used in bulk ATPase assays: in all molecules, ones that do not form the intact complex or do not rotate are included. Due to the same reason, the

[ATP] range at which the accelerated rotations are observed in the bulk ATPase measurements is wider than that in single-molecule experiments; the peak is shifted to a higher concentration than that in single-molecule experiments.

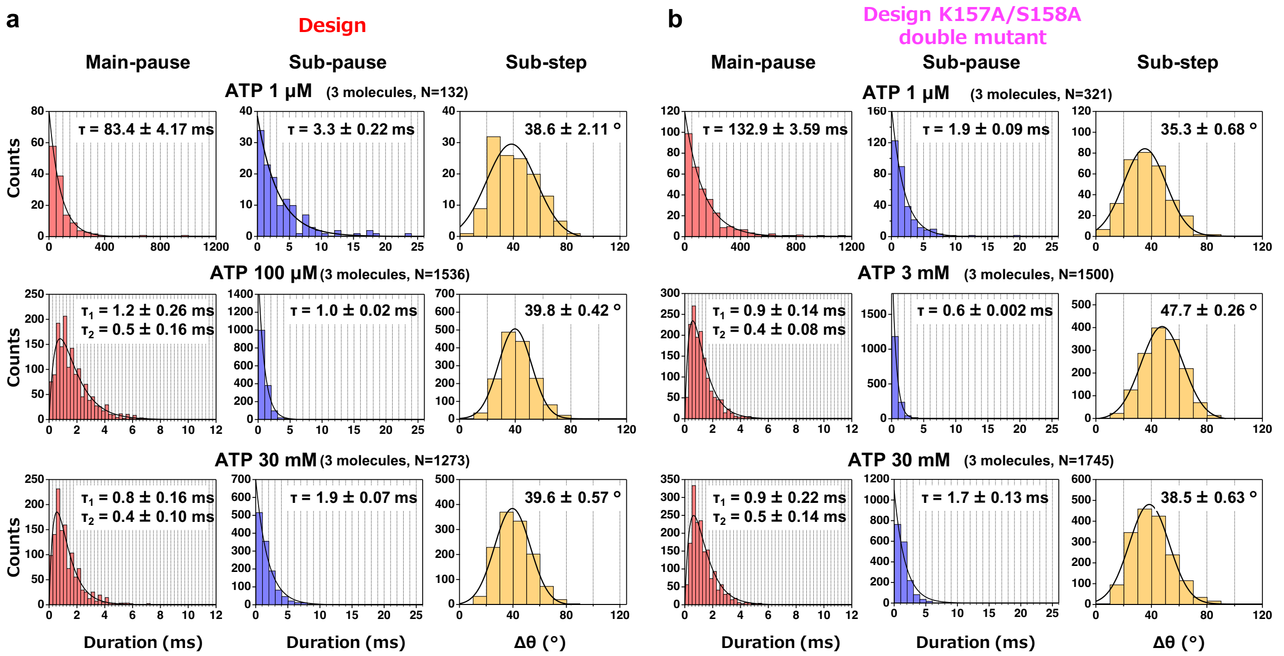

Supplementary Fig. 14: Duration time distributions with estimated time constants for main- and sub-pauses and Δθ for the designed V_1 and the design double mutant K157A/S158A. Duration time distribution for main- and sub-pauses and angle difference (Δθ) from main- to sub-pause for the design (**a**) and the design double mutant K157A/S158A (**b**). For the main-pause at 1 μM ATP, the distributions were fitted with a single-exponential decay function: constant × exp($-t/\tau$). For the main-pause at 100 μM, 3 mM and 30 mM ATP, the distributions were fitted with a double-exponential decay functions assuming two consecutive first-order reactions: constant × [exp($-t/\tau_1$) - exp($-t/\tau_2$)]. For the sub-pause at all [ATP]s, the distributions were fitted with a single-exponential decay function: constant × exp($-t/\tau$). The presented histograms were plotted by analyzing the all detected pauses for all molecules we used for the dwell time analysis at each ATP concentration. The distributions of Δθ were fitted with Gaussian function. The Δθ values of the designed V_1 are similar with that (approximately 40°) of the wildtype. All fittings were carried out for the values at the middle of the histogram bars, using the least-square fitting method. The distributions for individual molecules are shown in Supplementary Fig. 15.

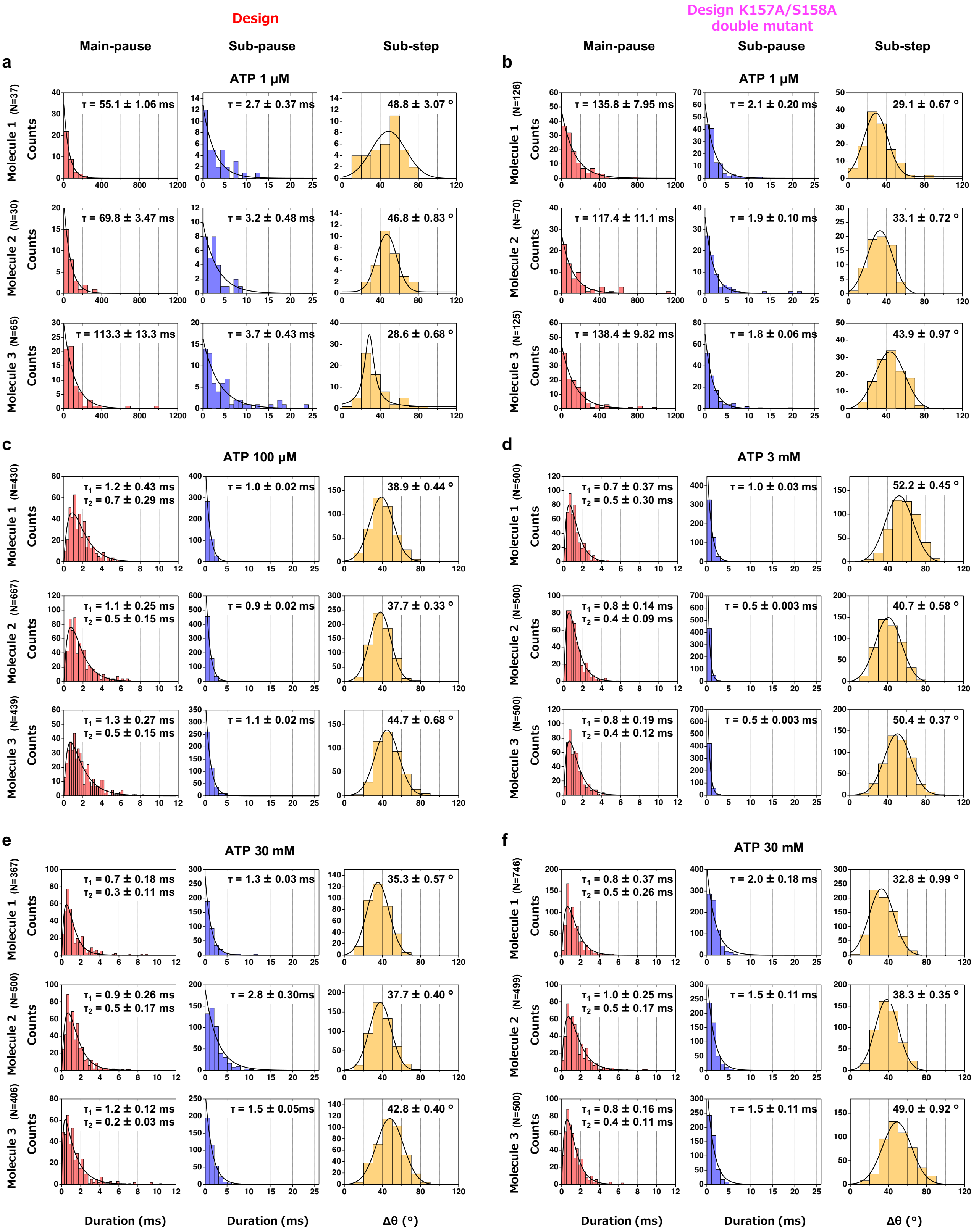

Supplementary Fig. 15: Duration time distributions with estimated time constants for main- and sub-pauses and Δθ for individual molecules. Duration time distribution for main- and sub-pauses

and angle difference (Δθ) from main- to sub-pause at (**a**) 1 μM ATP, (**c**) 100 μM ATP, and (**e**) 30 mM ATP for individual molecules of the design and at (**b**) 1 μM ATP, (**d**) 3 mM ATP, and (**f**) 30 mM ATP for individual molecules of the design double mutant K157A/S158A. The fittings were carried out using the same procedure used in Supplementary Fig. 14. The distributions for all molecules used for the dwell time analysis are shown in Supplementary Fig. 14.

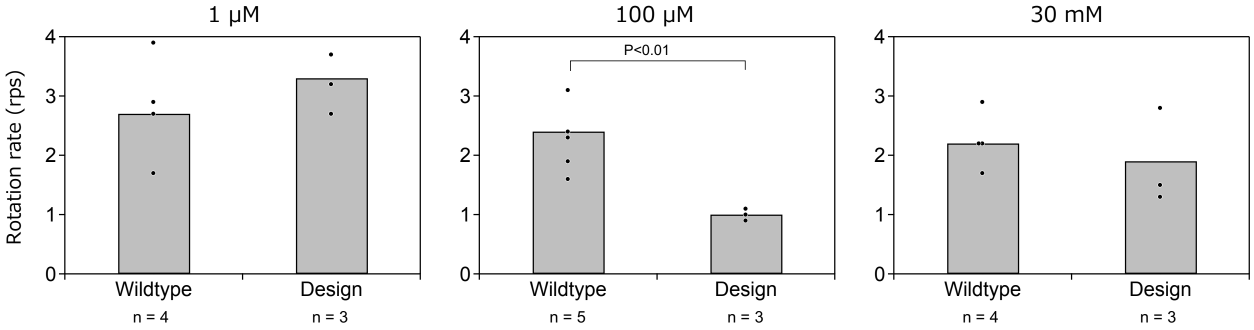

Supplementary Fig. 16: Comparisons for the sub-pause time constants between the wild-type and designed V_1 at several [ATP]s. The time constants were obtained from the duration time distributions in Supplementary Fig. 14 and 15. The time constants obtained from the data for all molecules and the data for individual molecules (n indicates the number of analyzed molecules) are shown by gray bars and black dots, respectively. By Welch's two-tailed t-tests, a significant difference (95% confidence interval, $t(4)=4.834$, $p=0.0084$) was found between the wild-type and designed V_1 at 100 μM ATP but not at 1 μM and 30 mM ATP. Hence, the sub-pause time constant of the designed V_1 significantly decreases at a certain range of [ATP]s, compared to the wild type.

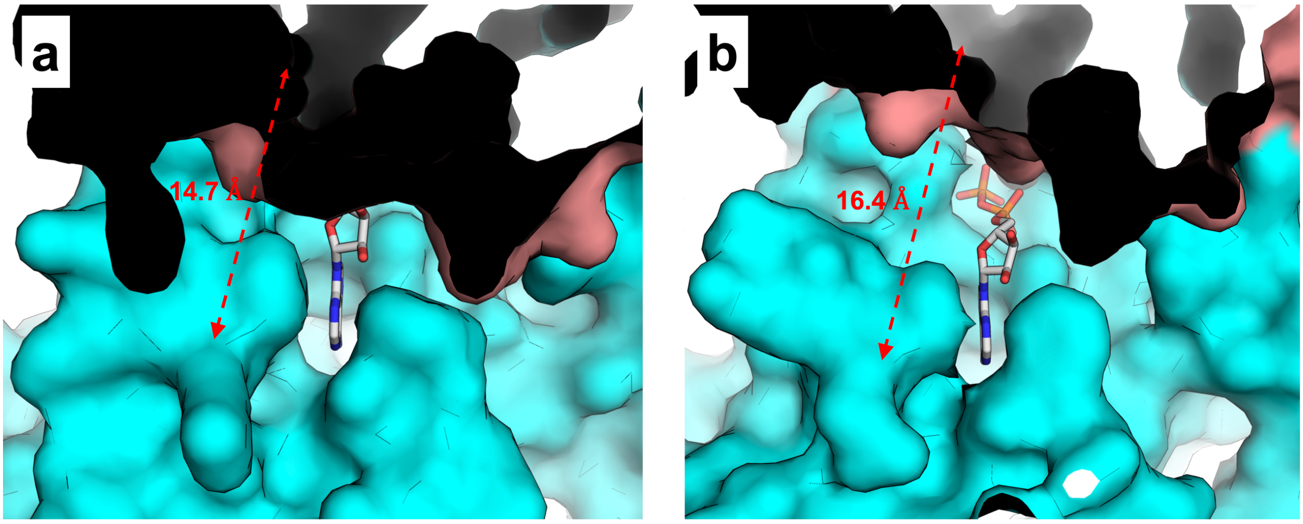

Supplementary Fig. 17: Structural comparison between the catalytic site in the presence and absence of an ADP molecule at the neighboring designed site. a, The catalytic site formed at the interface between the chain C (the A-subunit) and F (the designed B-subunit) in $A_3(De)_3\_(ADP)_{3cat,1non\text{-}cat}$, when no nucleotide is bound with the neighboring designed site. The distance between Cα atoms of F425 in chain C and S349 in Chain F are shown in red. **b**, The catalytic site formed at the interface between the chain I (the A-subunit) and L (the designed B-subunit) in $A_3(De)_3\_(ADP)_{3cat,2non\text{-}cat}$, when an ADP molecule is bound with the neighboring design site. The distance between Cα atoms of F425 in chain I and S349 in Chain L are shown in red. The A- and B-subunits are shown in cyan and black, respectively. Interactions of an ADP molecule with the catalytic site in (**b**) are more decreased compared with that in (**a**).

Supplementary Table 1: Number of molecules used for measurement of average rotation rates in the single-molecule experiments.

[ATP] (μM)	Number of molecules		
	Designed V_1	Designed V_1 K157Q	Designed V_1 K157A/T158A
1	7	3	5
10	3	3	6
30	4	4	4
100	8	4	5
300	3	3	4
1000	4	5	4
3000	4	6	9
10000	3	5	5
30000	5	5	7

Supplementary Table 2: Rosetta ddG score calculations to evaluate ATP binding ability of the designed V_1 and its mutants.

The adenine ring of the ADP was bound to V_1 at the interface between the A- and designed B-subunits. Rosetta ddG score calculations to evaluate ATP binding ability of the designed V_1 and its mutants were carried out, using the A- and B- subunit dimer with an ATP molecule at the designed interface. The structure models with an ATP used for the calculations were prepared by replacing the ADP at the interface between the chain C and D of $A_3(De)_3$_(ADP・Pi)$_{1cat}$(ADP)$_{2cat,2non\text{-}cat}$ to an ATP. The calculations were performed 10 times independently, and averaged ddG scores and their standard deviations are shown.

	ddG score
Design	-17.9 ± 0.4
Design K157Q	-15.6 ± 0.5
Design K157A/S158A	-14.4 ± 0.4

Supplementary Table 3: Comparison of rotation rates estimated from the slope in the time course of rotation and those estimated from the dwell time constants for the main- and sub-pauses.

	[ATP] (μM)	Rotation rate (rps) estimated from the slope in the time course of rotation	Rotation rate (rps) estimated from the dwell time constants
Designed V_1	1	3.0	3.8
	100	113.9	123.3
	30000	118.0	107.1
Designed V_1 K157A/S158A	1	2.2	2.5
	3000	160.6	177.3
	30000	135.7	108.4

Supplementary Table 4: Structural comparison of the catalytic interfaces between the nucleotide-free A_3B_3 complex of the wild-type (3VR2) and designed V_1 ($A_3(De)_3\_(ADP)_{3cat,1non\text{-}cat}$ and $A_3(De)_3\_(ADP)_{3cat,2non\text{-}cat}$).

RMSD values (Å) calculated by MICAN[1] are shown. Empty, Bindable and Bound represent conformational states of the catalytic interface defined in the Arai *et al*[5]. The bold numbers indicate the RMSD values for the catalytic interface with the largest conformational change between $A_3(De)_3\_(ADP)_{3cat,1non\text{-}cat}$ and $A_3(De)_3\_(ADP)_{3cat,2non\text{-}cat}$. One of the catalytic interfaces in the design complex changes to more open conformation upon binding a nucleotide at the next designed interface. Note that the RMSD values for chain BE in $A_3(De)_3\_(ADP)_{3cat,1non\text{-}cat}$ in parentheses are probably underestimated, since the part of C-terminal helical domain of chain B is deleted in the structural model because of the unclear density.

Catalytic Interface	State	$A_3(De)_3\_(ADP)_{3cat,1non\text{-}cat}$			$A_3(De)_3\_(ADP)_{3cat,2non\text{-}cat}$		
		Chain AD	Chain BE	**Chain CF**	Chain GJ	Chain HK	**Chain IL**
Wildtype, 3VR2 Chain AD	Empty	2.01	(2.62)	**3.47**	2.23	2.77	**2.87**
Wildtype, 3VR2 Chain BE	Bindable	1.98	(1.47)	**2.80**	1.42	1.85	**2.05**
Wildtype, 3VR2 Chain CF	Bound	2.62	(2.55)	**1.63**	3.19	2.48	**2.07**

Supplementary Table 5: Data collection and refinement statistics of crystal structures

	$A_3(De)_3$_empty<br>PDB: 8IGU	$A_3(De)_3$_(ADP·Pi)$_{1cat}$(ADP)$_{2cat,2non\text{-}cat}$<br>PDB: 8IGV	$A_3(De)_3$_(ADP)$_{3cat,1non\text{-}cat}$<br>$A_3(De)_3$_(ADP)$_{3cat,2non\text{-}cat}$<br>PDB: 8IGW
**Data collection**			
**Space group**	$P2_1$	$P2_1$	$P2_1$
**Cell dimensions**			
a, b, c **(Å)**	122.45, 122.65, 128.70	119.67, 126.74, 123.65	179.41, 125.08, 180.91
**α, β, γ (°)**	90.0, 90.7, 90.0	90.0, 93.9, 90.0	90.0, 93.8, 90.0
**Wavelength**	1.100	1.100	1.100
**Resolution (Å)**	44.64 – 2.77<br>(2.82 – 2.77)	48.38 – 3.15<br>(3.23 – 3.15)	48.92 – 4.20<br>(4.32 – 4.20)
R_{merge}	0.09 (0.88)	0.21 (1.11)	0.24 (1.07)
R_{pim}	0.040(0.380)	0.087(0.490)	0.095(0.423)
$I/\sigma I$	14.2 (2.1)	7.7 (2.0)	7.2 (2.1)
$C/C_{1/2}$	0.998(0.728)	0.984(0.569)	0.994(0.764)
**Completeness (%)**	100.0 (100.0)	100.0 (100.0)	99.9 (100.0)
**Redundancy**	7.0 (7.3)	7.1 (6.2)	7.5 (7.4)
**Refinement**			
**Resolution (Å)**	44.64 - 2.77	48.38 – 3.15	48.92 – 4.20
**No. reflections**	96799	63799	58615
R_{work}/R_{free}	0.216/0.262	0.211/0.268	0.242/0.281
**No. atoms**			
**Protein**	24039	24005	47540*
**Ligand/ion**	0	145	252
**Water**	253	117	0
**B-factors**			
**Protein**	74.60	56.39	153.35
**Ligand/ion**	-	60.53	174.87
**Water**	55.39	35.35	-
**R.m.s. deviations**			
**Bond length (Å)**	0.005	0.005	0.003
**Bond angles (°)**	0.978	0.939	0.651
**Ramachandran#**			
**favored**	96.06	96.53	95.75
**allowed**	3.91	3.44	4.07
**outeliers**	0.03	0.03	0.18

A single crystal was used to obtain each data set. Values in parentheses are for highest-resolution shell.

*Two $A_3(De)_3$ molecules in the asymmetric unit. In the structural models of $A_3(De)_3$_$(ADP)_{3cat,1non\text{-}cat}$ and $A_3(De)_3$_$(ADP)_{3cat,2non\text{-}cat}$, the electron density of the C-terminal domain of chain B in $A_3(De)_3$_$(ADP)_{3cat,1non\text{-}cat}$ is not clearly observed because of the unclear electron density. Although the resolution of these structures is relatively low (4.2 Å), the densities for the main chain Cα-trace and bound ADPs were clearly observed (Supplementary Fig. 8 and Extended Data Fig 1 and 2). Small extra density is observed at the designed sites in chain F in $A_3(De)_3$_$(ADP{\cdot}Pi)_{1cat}$ $(ADP)_{2cat,2non\text{-}cat}$, chain E in $A_3(De)_3$_$(ADP)_{3cat,1non\text{-}cat}$ and chain K in $A_3(De)_3$_$(ADP)_{3cat,2non\text{-}cat}$. These possibly correspond to a phosphate based on the density size and position, but attempts to place a phosphate resulted in severe crashing. We did not place any molecules at these sites due to the uncertainty of model.

[#] As defined by Molprobity

Supplementary Text

1. Designed B-subunit monomer binds to nucleotide in solution

To evaluate the ATP binding ability of De in the $A_3(De)_3$ complex, we introduced a double mutation in the A-subunit (K238A and T239A) to significantly impair ATP binding ability[6]. However, De did not form the $A_3(De)_3$ ring complex with the mutant A-subunit (Supplementary Fig. 7b). Therefore, we purified the designed B-subunit monomer (Supplementary Fig. 7c) and the nucleotide binding ability was indirectly evaluated by thermal shift[7] in circular dichroism spectroscopy in the presence or absence of nucleotides (Supplementary Fig. 9). The designed B-subunit monomer exhibited an increase of its melting temperature upon the addition of nucleotide, while the melting temperatures for the wild-type B-subunit monomer were almost the same in the presence and absence of nucleotides. These results strongly suggested that the designed B-subunit monomer has nucleotide binding ability.

2. Rosetta Scripts XML file for designing ATP binding site

Computational designs using Rosetta design software[8] were performed with the following XML file.

```
<ROSETTASCRIPTS>
    <SCOREFXNS>
        <SFXN weights=talaris2014 />
    </SCOREFXNS>
    <FILTERS>
        <Ddg name=ddg_calc scorefxn=SFXN jump=1 threshold=-8 repeats=5 repack=true />
    </FILTERS>
    <TASKOPERATIONS>
        <ReadResfile name=resfile filename="./3VR6_E_Ploop.resfile" />
        <LayerDesign name=layer_all layer=core_boundary_surface core=20 surface_E=70 surface_H=60
pore_radius=2.0 ignore_pikaa_natro=1 />
    </TASKOPERATIONS>
    <MOVERS>
        <AddOrRemoveMatchCsts name=cstadd cst_instruction=add_new/>
        <AddOrRemoveMatchCsts name=cstremove cst_instruction=remove/>
        <EnzRepackMinimize name=min_enz scorefxn_repack=SFXN scorefxn_minimize=SFXN design=1
minimize_bb=1 minimize_sc=1 minimize_rb=1 minimize_lig=1 cycles=20 task_operations=resfile,layer_all />
        <EnzRepackMinimize name=min scorefxn_repack=SFXN scorefxn_minimize=SFXN minimize_sc=1
minimize_rb=0 minimize_lig=1 design=0 repack_only=0 minimize_bb=0 cycles=20 />
        <Idealize name=ideal />
        <PredesignPerturbMover name=pre_min trans_magnitude=0.1 rot_magnitude=2.0 dock_trials=5000
/>
      </MOVERS>
      <PROTOCOLS>
            <Add mover_name=ideal />
            <Add mover_name=cstadd/>
            <Add mover_name=pre_min/>
            <Add mover_name=min_enz />
            <Add mover_name=cstremove/>
            <Add mover_name=min/>
            <Add filter_name=ddg_calc />
      </PROTOCOLS>
</ROSETTASCRIPTS>
```

Supplementary Data Figures

Supplementary Fig. 5a

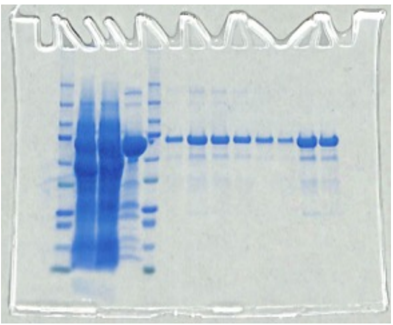

Supplementary Fig. 5b

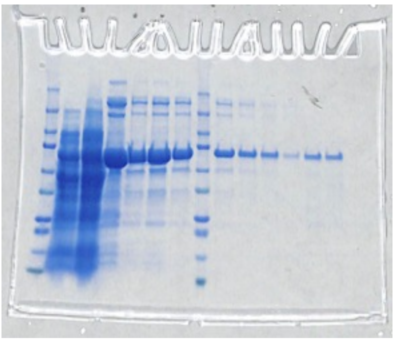

Supplementary Fig. 7a

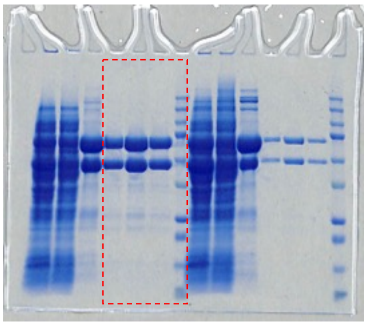

Supplementary Fig. 7b

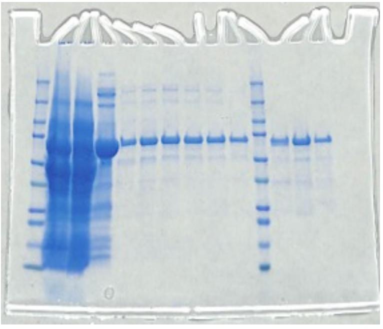

Supplementary Fig. 7c

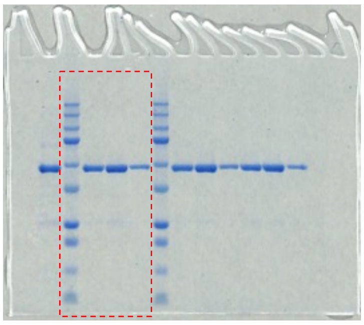

Supplementary Fig. 7d

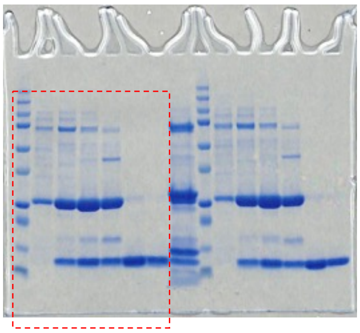

Supplementary Fig. 7e

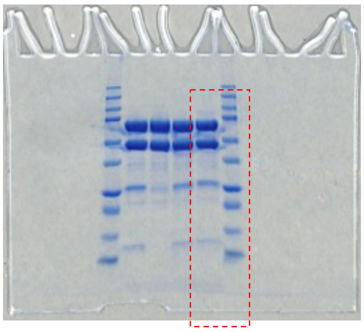

Supplementary References

1. Minami, S., Sawada, K. & Chikenji, G. MICAN : a protein structure alignment algorithm that can handle Multiple-chains, Inverse alignments, Cαonly models, Alternative alignments, and Non-sequential alignments. *BMC Bioinformatics* **14**, 24 (2013).
2. Iida, T. et al. Single-molecule analysis reveals rotational substeps and chemo-mechanical coupling scheme of Enterococcus hirae V1-ATPase. *J Biol Chem* **294**, 17017-17030 (2019).
3. Minagawa, Y. et al. Basic Properties of Rotary Dynamics of the Molecular Motor *Enterococcus hirae* V_1-ATPase. *J Biol Chem* **288**, 32700-32707 (2013).
4. Kobayashi, R., Ueno, H., Li, C.-B. & Noji, H. Rotary catalysis of bovine mitochondrial F_1-ATPase studied by single-molecule experiments. *Proceedings of the National Academy of Sciences* **117**, 1447 (2020).
5. Arai, S. et al. Rotation mechanism of Enterococcus hirae V1-ATPase based on asymmetric crystal structures. *Nature* **493**, 703-7 (2013).
6. Imamura, H., Funamoto, S., Yoshida, M. & Yokoyama, K. Reconstitution in vitro of V1 complex of Thermus thermophilus V-ATPase revealed that ATP binding to the A subunit is crucial for V1 formation. *J Biol Chem* **281**, 38582-91 (2006).
7. Greenfield, N.J. Using circular dichroism collected as a function of temperature to determine the thermodynamics of protein unfolding and binding interactions. *Nat Protoc* **1**, 2527-35 (2006).
8. Leaver-Fay, A. et al. ROSETTA3: an object-oriented software suite for the simulation and design of macromolecules. *Methods Enzymol* **487**, 545-74 (2011).
