# Supplementary material for: Design of allosteric sites into rotary motor V1-ATPase by restoring lost function of pseudo-active sites
Source: Nat Chem. 2023 Jul 6;15(11):1591–8. doi: 10.1038/s41557-023-01256-4 (PMC10624635; doi:10.1038/s41557-023-01256-4)
Supplement: Supplementary file 2 — Reporting Summary [file 41557_2023_1256_MOESM2_ESM.pdf]

nature research

Corresponding author(s): Takahiro Kosugi and Nobuyasu Koga

Last updated by author(s): Apr 14, 2023

# Reporting Summary

Nature Research wishes to improve the reproducibility of the work that we publish. This form provides structure for consistency and transparency in reporting. For further information on Nature Research policies, see our Editorial Policies and the Editorial Policy Checklist.

## Statistics

For all statistical analyses, confirm that the following items are present in the figure legend, table legend, main text, or Methods section.

| n/a | Confirmed | |
|---|---|---|
| ☐ | ☒ | The exact sample size (*n*) for each experimental group/condition, given as a discrete number and unit of measurement |
| ☐ | ☒ | A statement on whether measurements were taken from distinct samples or whether the same sample was measured repeatedly |
| ☐ | ☒ | The statistical test(s) used AND whether they are one- or two-sided<br>*Only common tests should be described solely by name; describe more complex techniques in the Methods section.* |
| ☒ | ☐ | A description of all covariates tested |
| ☒ | ☐ | A description of any assumptions or corrections, such as tests of normality and adjustment for multiple comparisons |
| ☐ | ☒ | A full description of the statistical parameters including central tendency (e.g. means) or other basic estimates (e.g. regression coefficient) AND variation (e.g. standard deviation) or associated estimates of uncertainty (e.g. confidence intervals) |
| ☐ | ☒ | For null hypothesis testing, the test statistic (e.g. *F*, *t*, *r*) with confidence intervals, effect sizes, degrees of freedom and *P* value noted<br>*Give P values as exact values whenever suitable.* |
| ☒ | ☐ | For Bayesian analysis, information on the choice of priors and Markov chain Monte Carlo settings |
| ☒ | ☐ | For hierarchical and complex designs, identification of the appropriate level for tests and full reporting of outcomes |
| ☒ | ☐ | Estimates of effect sizes (e.g. Cohen's *d*, Pearson's *r*), indicating how they were calculated |

*Our web collection on statistics for biologists contains articles on many of the points above.*

## Software and code

Policy information about availability of computer code

| | |
|---|---|
| Data collection | Rosetta software suite 3 was used for protein design calculations. Amber14 software suite was used for MD simulations. JASCO SpectraManager software v2 was used for CD. |
| Data analysis | Analyses on protein structure were carried out Rosetta software suite 3 and MICAN Version 2018.04.05.<br>Conformations of ATP molecules were generated by BCL Version 3.4.0.<br>Trajectories of MD simulations were analyzed by ccptraj module in Amber14.<br>Thermal denaturation data by CD were fit with a sigmoidal function using JASCO SpectraManager software v2.<br>Single molecule images were analyzed by a custum-made plugin of the Image J software which was developed in the paper (K. Adachi. et al., Cell. 2007).<br>All crystal structure analyses were done as described in the methods section with the following programs: PHENIX 1.14 or 1.18, REFMAC5 (CCP4 Suite 7.0 or 7.1), COOT Version 0.8.<br>Figures were prepared by the following programs: PyMOL Version 2.1, CueMol2 Version 2.2.3.443, Chimera Version 1.13.1. |

For manuscripts utilizing custom algorithms or software that are central to the research but not yet described in published literature, software must be made available to editors and reviewers. We strongly encourage code deposition in a community repository (e.g. GitHub). See the Nature Research guidelines for submitting code & software for further information.

## Data

Policy information about availability of data

All manuscripts must include a data availability statement. This statement should provide the following information, where applicable:

- Accession codes, unique identifiers, or web links for publicly available datasets
- A list of figures that have associated raw data
- A description of any restrictions on data availability

Coordinates and structure factors for the designed V1-ATPase were deposited in the Protein Data Bank under the accession number 8IGU [https://doi.org/10.2210/pdb8IGU/pdb] , 8IGV [https://doi.org/10.2210/pdb8IGV/pdb] and 8IGW [https://doi.org/10.2210/pdb8IGW/pdb]. The designed model structure and MD-related data are provided in Supplementary Data File.

# Field-specific reporting

Please select the one below that is the best fit for your research. If you are not sure, read the appropriate sections before making your selection.

☒ Life sciences ☐ Behavioural & social sciences ☐ Ecological, evolutionary & environmental sciences

For a reference copy of the document with all sections, see nature.com/documents/nr-reporting-summary-flat.pdf

# Life sciences study design

All studies must disclose on these points even when the disclosure is negative.

| | |
|---|---|
| Sample size | Computational designs that passed selection criteria were experimentally tested. Sample sizes for various experimental assays are reported in the main text, Supplementary information, and Source Data file. Each sample size of single-molecule experiments was determined according to previous study (T. Iida. et al., J. Biol. Chem. 2019) |
| Data exclusions | For analyses of single-molecule experiments, image data that does not show symmetrical and smooth rotations were excluded. |
| Replication | All reported experiments, including sample purification, activity assay, crystallization, and single-molecule experiments were replicated at least twice. |
| Randomization | This study did not include experiments with experimental group allocation. Therefore, no randomization methods were used in this study. |
| Blinding | This study did not include experiments with experimental group allocation. Therefore, no blinded experiments were used in this study. |

# Reporting for specific materials, systems and methods

We require information from authors about some types of materials, experimental systems and methods used in many studies. Here, indicate whether each material, system or method listed is relevant to your study. If you are not sure if a list item applies to your research, read the appropriate section before selecting a response.

## Materials & experimental systems

| n/a | Involved in the study |
|---|---|
| ☒ | ☐ Antibodies |
| ☒ | ☐ Eukaryotic cell lines |
| ☒ | ☐ Palaeontology and archaeology |
| ☒ | ☐ Animals and other organisms |
| ☒ | ☐ Human research participants |
| ☒ | ☐ Clinical data |
| ☒ | ☐ Dual use research of concern |

## Methods

| n/a | Involved in the study |
|---|---|
| ☒ | ☐ ChIP-seq |
| ☒ | ☐ Flow cytometry |
| ☒ | ☐ MRI-based neuroimaging |
